# Mitochondria-targeting fluorescent molecules for high efficiency cancer growth inhibition and imaging†
†Electronic supplementary information (ESI) available. See DOI: 10.1039/c9sc01410a


**DOI:** 10.1039/c9sc01410a

**Published:** 2019-07-08

**Authors:** Hao Chen, Jing Wang, Xin Feng, Mark Zhu, Simon Hoffmann, Alex Hsu, Kun Qian, Daijuan Huang, Feng Zhao, Wei Liu, Huimao Zhang, Zhen Cheng

**Affiliations:** a Department of Radiology , The First Hospital of Jilin University , Changchun , 130021 , China . Email: huimaozhanglinda@163.com; b Molecular Imaging Program at Stanford (MIPS) , Bio-X Program , Department of Radiology , Canary Center at Stanford for Cancer Early Detection , Stanford University , California 94305-5344 , USA . Email: zcheng@stanford.edu; c Center for Molecular Imaging Research , Shanghai Institute of Materia Medica , Chinese Academy of Sciences , Shanghai , 201203 , China; d The College of Veterinary Medicine , Jilin University , Changchun , 130021 , China

## Abstract

Fluorescent mitochondria-accumulating delocalized lipophilic cations (DLCs) for cancer therapy have drawn significant attention in the field of cancer theranostics. One of the most promising fluorescent DLCs, F16, can selectively trigger the apoptosis and necrosis of cancer cells, making it an attractive targeted theranostic drug candidate. However, it suffers from low clinical translation potential, largely due to its inefficient anti-cancer activity (IC_50_ in the μM range) and poorly understood structure–activity relationship (SAR). In this report, eleven indole-ring substituted F16 derivatives (F16s) were synthesized. Among these derivatives, **5BMF** was identified as a highly effective theranostic agent, with *in vitro* studies showing a low IC_50_ of ∼50 nM (to H2228 cells) and high cancer to normal cell selectivity index of 225. *In vivo* studies revealed that tumors treated with **5BMF** were significantly suppressed (almost no growth over the treatment period) compared to the PBS treated control group, and also no obvious toxicity to mice was found. In addition, the tumor imaging capability of **5BMF** was demonstrated by *in vivo* fluorescence imaging. Finally, we report for the first time a proposed SAR for F16 DLCs. Our work lays down a solid foundation for translating **5BMF** into a novel and highly promising DLC for cancer theranostics.

## Abstract 2

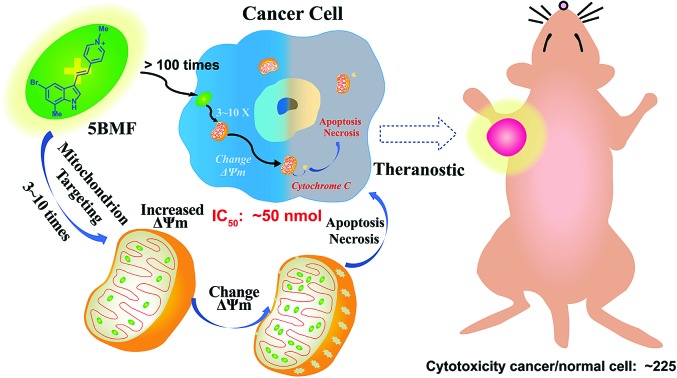
 **5BMF** is a new fluorescent mitochondria-accumulating delocalized lipophilic cations [DLC] that boasts significantly increased anti-cancer effects and low toxicity in comparison to previous DLCs, addressing current hurdles in DLC clinical translation.

## Introduction

Development of chemotherapeutic drugs for cancer-based on mitochondria-targeting molecules has been an intensely explored field.^1^ Delocalized lipophilic cations (DLCs) can accumulate in cancer cell mitochondria with >100 fold selectivity over normal cell mitochondria^2^ because of the natural mitochondrial membrane potential difference between cancer cells (Δ*Ψ*_c_, ∼–220 mV)^3^ and normal cells (Δ*Ψ*_n_, ∼–140 mV),^4^,^5^ representing one class of the most attractive mitochondria-targeting anti-cancer agents. Many DLCs also function as fluorescent dyes^6^,^7^ due to their rigid structure, such as the first DLC discovered – rhodamine 123 (Fig. S1†).^8^–^10^ As a result, plenty of DLCs are naturally theranostic agents, having potential applications in chemotherapy, photothermal/photodynamic therapy, optical imaging and fluorescence image-guided surgery.^11^,^12^ Furthermore, some DLCs such as rhodamine 123 exhibit avidity to tumor hypoxia. When these mitochondrial-targeting agents are combined with 2-deoxy-d-glucose (2-DG, Phase II clinical trial), enhanced toxicity against drug-resistant tumors and improved normal cell compatibility can be achieved compared to either drug alone.^13^–^15^ Such new DLCs can offer a powerful regimen for cancer therapy.

**Scheme 1:**
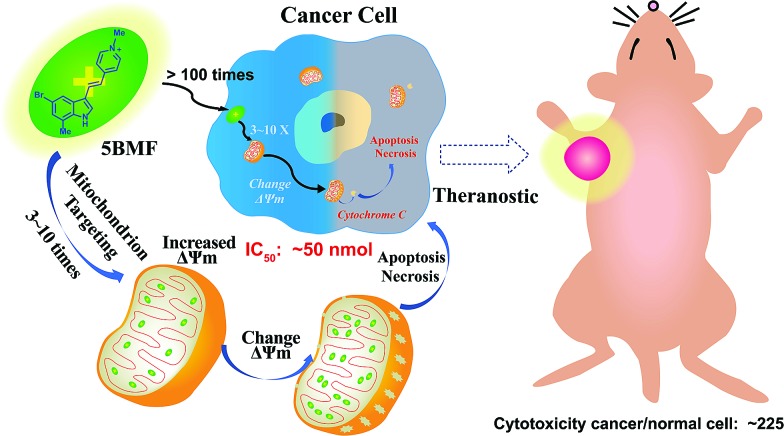
Mitochondria-targeting F16 derivative **5BMF** for high efficiency cancer treatment and imaging.

Currently, rhodamine 123, MKT-077, and F16 are the most representative DLCs (Fig. S1†), and they have drawn great interest for cancer drug discovery. MKT-077 is the first DLC tested in a clinical trial, though the study was halted at Phase II due to limited anti-tumor effect and mild nephrotoxicity.^10^,^16^,^17^ Another DLC, F16, was identified through a high-throughput chemical library screen. It selectively accumulates in the mitochondria of cancer cells, disrupting normal function by dissipating the proton gradient across the inner mitochondrial membrane (decrease of Δ*Ψ*_m_, Scheme 1), triggering apoptosis or necrosis depending on the genetic background.^18^ However, F16's therapeutic efficacy has often been thought to be insufficient for drug development,^19^ as previous studies on structural modifications of F16 resulted in a limited improvement on their anti-cancer activity.^20^,^21^ A general opinion arose that DLCs naturally have limited anti-cancer activity because they often fail to cause large disruption of mitochondrial membrane potential (decrease of ATP production) to kill cancer cells.^19^,^22^ As a result, DLCs have been used simply as a cargo group to deliver functional molecules to the mitochondria selectively.^19^,^23^–^25^

Herein, we synthesized a small library of F16 derivatives (F16s, 11 compounds), each with different substitutions at the indole position. Optical spectrum measurement studies indicated that they could be used for both *in vitro* and *in vivo* fluorescence imaging. *In vitro* cell studies of F16s showed strong anti-cancer cell activity, with an IC_50_ as low as ∼50 nM, and up to 225 fold higher selectivity for cancer cells over normal cells (Scheme 1). The most promising analog, **5BMF**, was chosen for further *in vivo* studies using non-small-cell lung cancer (NSCLC) mouse models. After 21 days of treatment, the tumor volume of the **5BMF** treated mice decreased dramatically compared to that of the control, with no obvious toxicity. Moreover, the *in vivo* fluorescence imaging results indicated that **5BMF** could also be used as a targeted fluorescent probe for tumor diagnosis and potentially image-guided surgery. This study reports the first high efficiency indole-substituted F16 developed as a DLC based mitochondria-targeting tumor theranostic agent, as well as the first proposal of the structure–activity relationship (SAR) of F16. The results of this work provide a new perspective to high-efficiency anti-cancer DLC development.

## Results and discussion

**Fig. 1:**
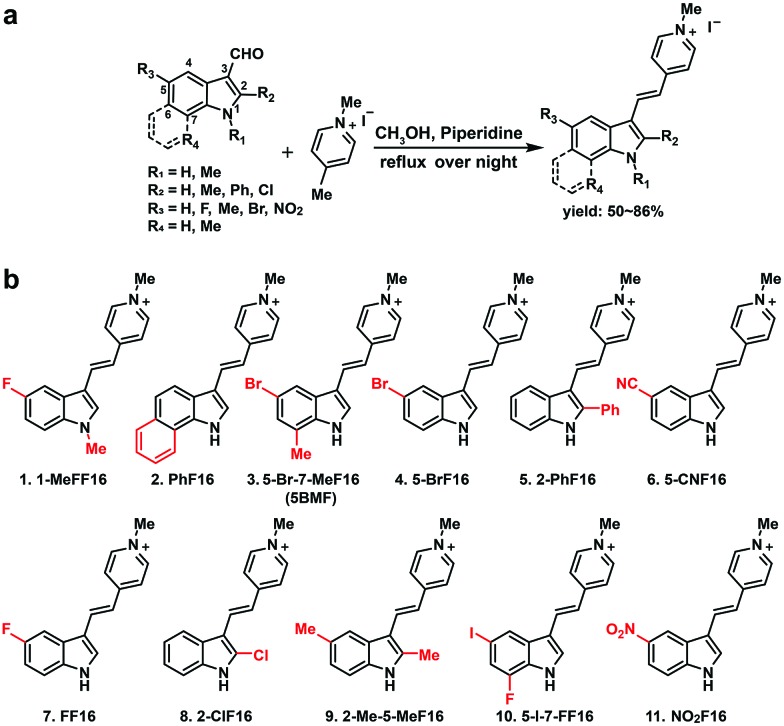
Synthesis of F16 derivatives (F16s) (a) and their chemical structures (b).

The key points for designing an effective F16 compound for cancer theranostic are: (1) a single positive charge on one part of the compound; (2) a whole-molecule π-conjugated system to spread the positive charge all over the compound; (3) to adjust the substituent group to fine-tune the charge distribution and polarity for efficient accumulation in mitochondria. F16 tautomerization studies show how positive charge spreads over the rigid structure (Fig. S2†). Positions 1, 2, 5, and 7 are the ideal positions to bear a positive charge (Fig. 1a). Changing the substituted group at those positions can affect the polarity and positive charge distribution of the compounds. These changes can also theoretically affect cross-membrane activity, resulting in further variations in anti-tumor activity. Keeping this hypothesis in mind, we designed and synthesized eleven F16 derivatives with methyl, phenyl, halogen, nitro, and nitrile substitutions at the 1, 2, 5, 6, and 7 positions of the indole ring according to a reported method,^21^ with yields between 50 and 86% (Fig. 1b). Recrystallization and semi-preparative HPLC purification methods were used to achieve a final purity for all F16 products of above 98% (Fig. S3†). The maximum solubility of F16s in water at room temperature is around 1 mM (Fig. S4†).

**Fig. 2:**
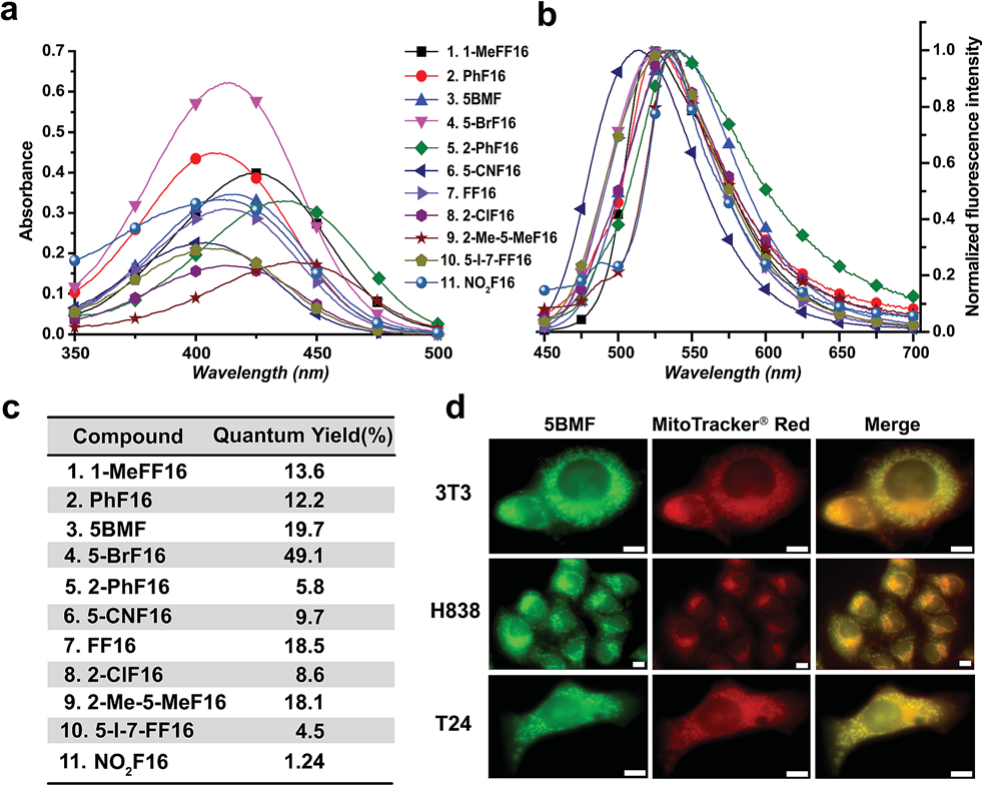
Optical properties of F16s. The absorption (a) and normalized emission (b) spectra of F16s were measured in water with a concentration of 31 μM and 7.8 μM, respectively (legend applies to both a and b). (c) F16s' relative quantum yields. EtOH as the solvent, compared with rhodamine 6G (QY: 95%). (d) Colocalization of **5BMF** (GFP channel) with mitochondrial specific probe MitoTracker® red (microscopy RFP channel). The Pearson's correlation coefficient (*γ*) for 3T3, H838 and T24 is 0.978, 0.924, and 0.929, respectively (calculated using ImageJ, JACoP plugin). 63× oil objective lens. White scale bar: 10 μm.

The absorbance, fluorescence, and photostability of all F16 derivatives were measured in aqueous solution with concentrations of 31 μM, 7.8 μM, and 10 μM, respectively. The absorption peaks of all F16s were around 425 nm, except compounds **5** and **9** for which they were about 450 nm. Compound **4** shows the greatest absorbance at 425 nm. All F16s' fluorescence peaks were around 525 nm (Fig. 2b). Quantum yields were measured in ethanol using rhodamine 6G as a reference. Compound **4** shows the highest quantum yield (49.1%). The quantum yield of compounds **3**, **7**, and **9** is around 20%, **1** and **2** around 12%, and **5**, **6**, **8**, **10**, and **11** under 10%. Compound **2**, **3**, **4**, **5**, **7**, and **11** have similar photostability to rhodamine 6G and MitoTracker® Green, while **1**, **8**, and **9** bleached quite easily (Fig. S5†).

R_1_ (Fig. 1a) methyl substituted **1** revealed the highest absorption (compared to **7**), but dramatically decreased photostability (Fig. S5,† comparing **1** with **7**). This implies the importance of the indole nitrogen alkyl substitution in the fluorescent dye structure design. R_2_ chlorinated and methylated **8** and **9** demonstrated poor photostability maybe because the R_2_ position of F16s is an electron-deficient position (Fig. S2†) which can easily react with an electron-rich group. In aqueous solution, under laser excitation, the electron-rich ^–^OH can attack the R_2_ position, destroy the delocalized π bond, and quench the fluorescence. Notably, R_3_ bromine substituted **4** showed double the molar absorptivity compared to fluorine substituted **7** (Fig. 2a). This implies that substitutions of different electronegativities can significantly impact the molar absorptivity, which may inspire the design of photoacoustic imaging probes and organic photoelectric materials.^26^

To test F16's mitochondrial targeting properties, the synthesized F16 derivatives were co-stained with MitoTracker® red. The results showed that all F16s were able to stain the mitochondria of all tested cell lines (Fig. S6†). As a representative, **5BMF** (**3**) colocalized with MitoTracker in NIH-3T3, H838, and T24 cell lines is shown in Fig. 2d. Its Pearson's correlation coefficient (*γ*) is 0.978, 0.924, and 0.929, respectively.^27^

**Fig. 3:**
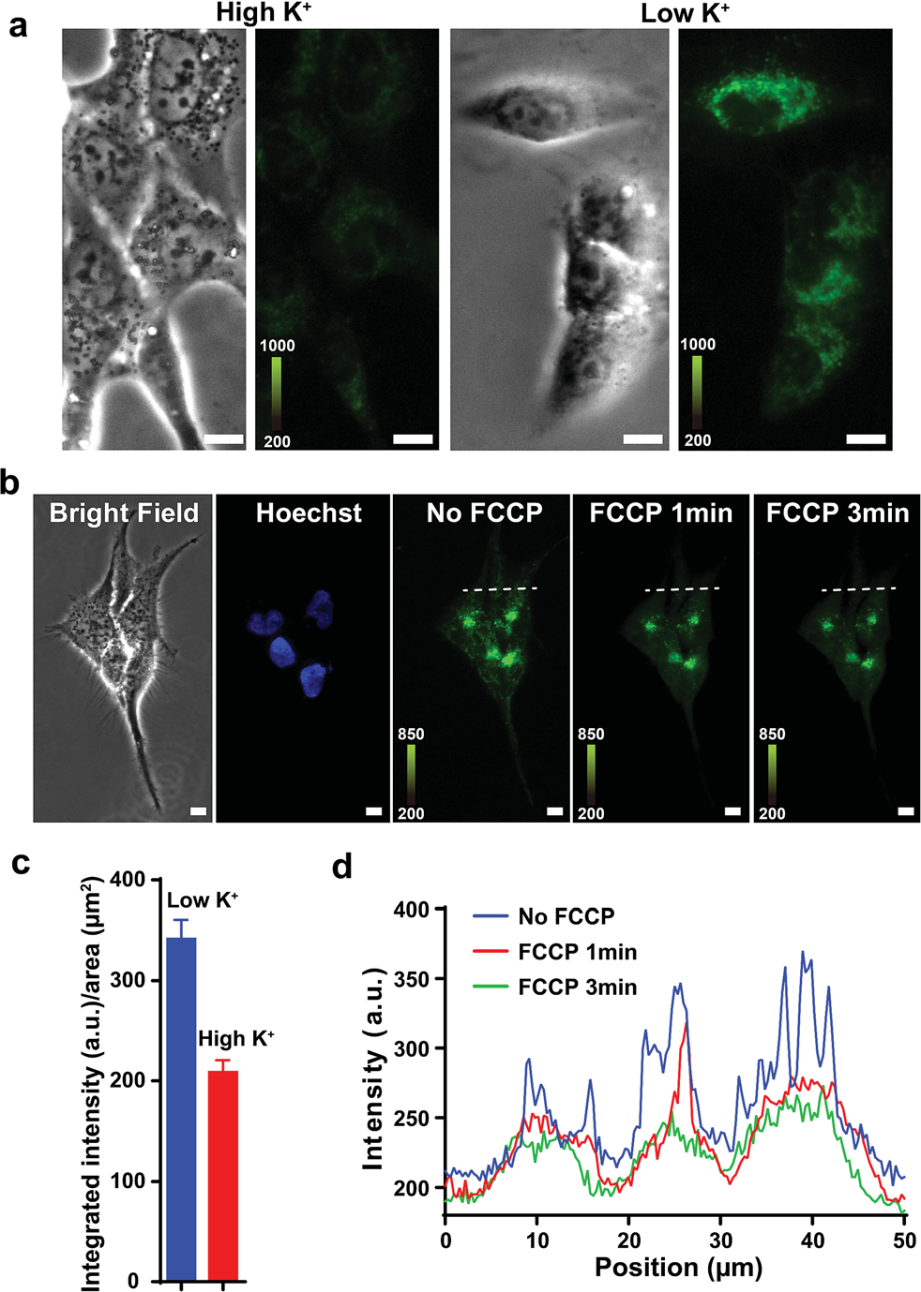
(a) Fluorescent signal in H838 cells' mitochondria in high K^+^ and low K^+^ buffers after incubation with **5BMF** for 30 min. Left: bright field image. Right: fluorescence image. (b) **5BMF** fluorescent signal in H838 cells' mitochondria before and after FCCP was added. From left to right, H838 cells bright field imaging, Hoechst staining of the nucleus, **5BMF** staining of mitochondria (GFP channel, green) without FCCP, and 5BMF staining after adding FCCP for 1 and 3 min. All images were taken with a 20× objective lens. (c) Mean fluorescence density of cells in low K^+^ and high K^+^ buffers. (d) Cross-sectional fluorescence intensity profiles taken along white-dashed lines of (b). White scale bar: 10 μm.

Previous studies show that F16 is driven into the mitochondria by their membrane potential (Δ*Ψ*_m_).^18^ To verify that F16 derivatives preserve the same property, H838 cells were preincubated in high K^+^ medium (137 mM) to depolarize the plasma membrane, then **5BMF** (3 μM) was added and incubated for 30 min. Compared to low K^+^ (3.6 mM) medium, high K^+^ medium H838 resulted in much less **5BMF** accumulation in mitochondria (Fig. 3a and c). In addition, when carbonyl cyanide *p*-(trifluoromethoxy)phenylhydrazone (FCCP), a protonophore that dissipates the Δ*Ψ*_m_, was added to cells preloaded with **5BMF**, the mitochondrial staining immediately decayed and diffused into the cytoplasm (Fig. 3b and d). As with other DLCs, the selectivity of F16s for mitochondria is driven by the negative transmembrane potential.^18^

**Fig. 4:**
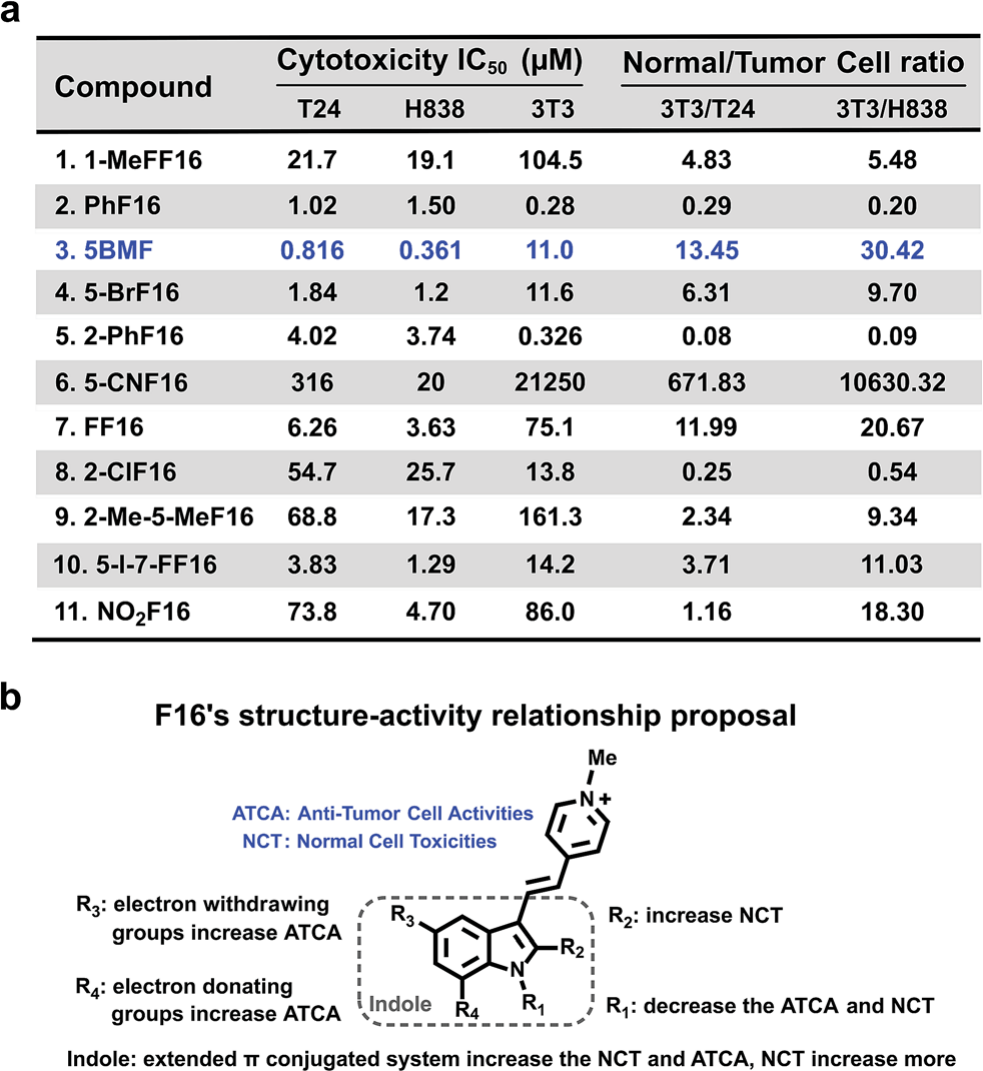
(a) F16s' cytotoxicity IC_50_ of T24, H838, and NIH-3T3 cell lines and their ratio. (b) F16s structure–anti-tumor activity relationship proposal.

The cytotoxicity of F16 derivatives was tested *in vitro* with the bladder cancer T24 cell, NSCLC H838 cell, and normal cell NIH-3T3. Six of the eleven F16s displayed a strong anti-tumor activity, with IC_50_ values within 0.36–6.2 μM (**2**, **3**, **4**, **5**, **7**, and **10**). Two of them showed selectivity indices larger than 10 (**3** and **7**). **5BMF** had the best anti-tumor activity and relatively high selectivity (Fig. 4a). The **5BMF**'s IC_50_ for T24 and H838 was 0.82 and 0.36 μM, compared to that of F16,^28^ which was 18.8 and 46.6 μM (Fig. S7†), 23 and 129 times lower, respectively. From the results of the *in vitro* study, we propose the SAR of F16s as follows (Fig. 4b): R_1_ substitution with the methyl group decreases anti-tumor cell activity (ATCA) and normal cell toxicity (NCT) (compared to **1** and **7**); R_2_ substitution increases NCT (compared to **5** and **8**); R_3_ substitution by an electron withdrawing group increases ATCA, and was the biggest factor in ATCA (compared to **4**, **6**, **7**, and **11**); R_4_ substitution by an electron donating group increases ATCA (compared to **3**, **4**, and **10**); indole ring conjugation with an extended π system increases the NCT and ATCA (compared to **2**, F16).

To further prove that an acceptor at the R_3_ position and a donor at the R_4_ position would increase the cell toxicity, another F16 analog **5-Cl-7-MeF16** with an acceptor at the R_3_ position (–Cl) and a donor group at the R_4_ position (–CH_3_) was synthesized (Fig. S8a†). Its cytotoxicity IC_50_ and normal cell to tumor cell ratio were also tested using T24, H838, and 3T3 cells (Fig. S8b†). Compared with **5BMF**, the **5-Cl-7-MeF16**'s ATCA is slightly better (T24: 0.390 μM; H838: 0.274 μM) but its NCT is higher (3T3: 0.964 μM). This demonstrates that an acceptor group at the R_3_ position and a donor group at the R_4_ position surely increase the ATCA. But suitable substituent groups are needed to maintain a good normal cell to tumor cell toxicity ratio.

For years, most of the research regarding F16 based anti-cancer agents targeted the *N*-methylpyridinium group for modifications (Fig. S1†). But changing the *N*-methylpyridinium's substituted position was found to have limited effects on increasing F16's anti-cancer activity.^20^ In addition, our previous study showed that conjugating triphenylphosphonium (TPP) salts (Fig. S1†) to the *N*-methylpyridinium position leads to anti-cancer activity loss.^29^ Thus, it seems that the indole ring plays a key role in determining F16's anti-tumor activity.

**Fig. 5:**
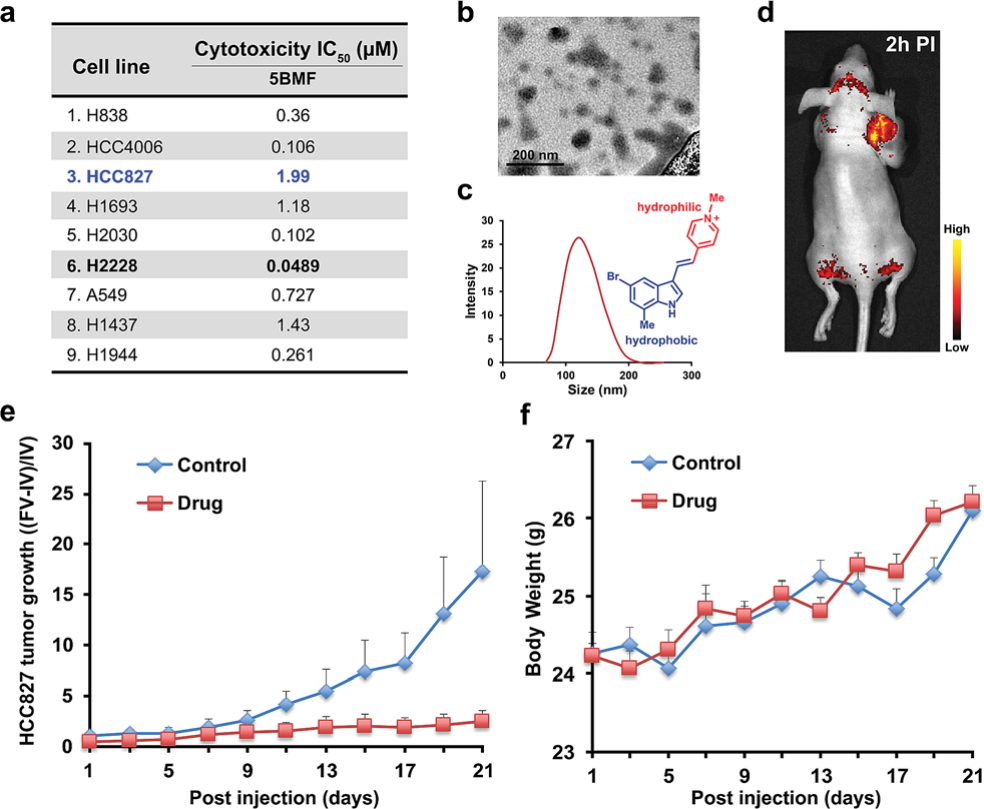
(a) **5BMF**'s IC_50_ of 9 lung cancer cell lines. (b) TEM image of **5BMF**, scale bar: 200 nm. (c) DLS curve of **5BMF** in PBS buffer, inset image: chemical structure of the amphiphilic **5BMF** with the hydrophilic *N*-methylpyridinium group (red) and hydrophobic indole group (blue). (d) A representative *in vivo* fluorescence image of a human lung cancer HCC827 bearing mouse 2 h post-injection (PI) of **5BMF** (15 mg kg^–1^, 0.306 M kg^–1^; for a 20 g mouse, inject 6.11 mM **5BMF** PBS solution 150 μL; fluorescence imaging was performed with 450 nm excitation light, and the emission light was collected using a 550 nm long-pass filter) (*n* = 4). (e) Effects of **5BMF** on the tumor growth of HCC827 in nude mice (*n* = 10 per group). FV, final volume. IV, initial volume. (f) Effects of **5BMF** on the tumor-bearing nude mice's body weight (*n* = 10 per group). The treatment was started when the tumors reached ∼3 mm in diameter (day 1). Control group: PBS. Drug group: **5BMF**, 15 mg kg^–1^, 0.306 M kg^–1^; for a 20 g mouse, inject 6.11 mM **5BMF** PBS solution 150 μL; IV injections were given on day 1, 3, 5, 7, 9, 11, 13, 15, 17, 19, and 21.

The NSCLC mouse model was then chosen as an especially valuable route for further *in vivo* study of F16s' anti-tumor activity. Lung cancer is the most common malignancy and is the leading cause of cancer-related deaths worldwide.^30^ 85% of lung cancers are NSCLC.^31^ For most patients with advanced or recurrent NSCLC, tumor recurrence and progression caused by drug resistance is a major cause of therapeutic failure.^32^ New anti-NSCLC drug development is urgent and imperative.^33^ Here, the cytotoxicity of the promising **5BMF** was tested with 9 NSCLC cell lines including H838, HCC4006, HCC827, H1693, H2030, H2228, A549, H1437, and H1944. **5BMF** showed impressively high ATCA in all these cell lines. In particular the H2228 cell line showed an IC_50_ of 48.9 nM, in addition to an anti-cancer cell to anti-normal cell (3T3, Fig. 4a) ratio around 225 (**Fig. 5a**).

The amphiphilic F16s were composed of the hydrophilic *N*-methylpyridinium group and hydrophobic indole group, which have the potential to self-assemble into nanoparticles in aqueous solution (**Fig. 5c**). **5BMF** was found to form a self-assembly of ∼120 nm nanoparticles in 1× pH 7.4 PBS from 5 mM to 0.125 mM concentration (Fig. 5b and c). And it decayed less than 20% in pH 3.0–10.0 buffers for 24 h, which demonstrated its stability at different pH (Fig. S9†). Usually, ∼120 nm nanoparticles could accumulate in solid tumors by the enhanced permeability and retention (EPR) effect.^34^ In order to verify **5BMF**'s tumor accumulation ability, the well-explored HCC827 cell was chosen for *in vivo* imaging study.^35^,^36^ **Fig. 5d** shows typical fluorescence images taken 2 h post intravenous injection (PI) of 15 mg kg^–1^**5BMF** into nude mice bearing subcutaneous HCC827 tumor. The tumor was clearly visualized from the surrounding background tissue with a tumor to background ratio of ∼2, which was sufficient for fluorescence image-guided surgery. Quantitative analysis of *ex vivo* tumor imaging at 40 min and 2 h PI is shown in Fig. S10.† The tumor, kidney, bone, stomach, bowel, pancreas, and skin accumulated more **5BMF** compared with the liver, spleen, heart, lung, and brain (Fig. S10a†). The tumor to most normal organ ratios (2 h PI, ∼2 to ∼7, Fig. S10b†) were high, and especially high tumor/lung ratios were observed (∼7, Fig. S10b†), implying potential low lung tissue toxicity and use for tumor detection and fluorescence image-guided lung cancer surgery applications.

In order to prove the anti-tumor effect of **5BMF** persuasively, the highest IC_50_ (**Fig. 5a**) HCC827 cell was chosen for the further *in vivo* treatment study. The treatment was started when the subcutaneous HCC827 tumors reached ∼3 mm in diameter (day 1). The dosage and administration frequency were 15 mg kg^–1^, IV injections on day 1, 3, 5, 7, 9, 11, 13, 15, 17, 19, 21, which were referenced from a MKT-077 study.^37^**Fig. 5e** shows the treatment response of the HCC827 xenograft to intravenous (IV) injection with **5BMF**. As indicated by the mean tumor growth rate in both control and treated groups, the inhibitory tumor growth effects for **5BMF** appeared from the 9^th^ day after treatment and exhibited significant ATCA at 21 days PI (**Fig. 5e**). The tumor growth of the **5BMF** treated group (drug group) was suppressed dramatically compared to the PBS treated group (control group), with a statistically significant result (*p* = 0.0426, statistical comparison at the 21^st^ day of the treatment). Approximately the mean tumor volume of the drug group increased ∼2 times after the 21 day-treatment period, while that of the control group increased ∼16 times. Meanwhile the body weight between the control and treated groups showed no significant difference (*p* = 0.477) (**Fig. 5f**), implying low *in vivo* toxicity of **5BMF**. Tumor pathologic section H & E staining results displayed that **5BMF** caused tumor inflammatory cell infiltration, fibrous tissue proliferation, and cell apoptosis (Fig. S11†) similar to the parent compound F16.^28^ The major organs' histologic examination results did not reveal any significant microscopic lesions in the **5BMF** treated mouse group over four weeks, compared to the control group (Fig. S12†).

## Conclusions

In summary, this study demonstrates that various substitutions in F16's indole ring significantly affect the absorbance, fluorescence and anti-tumor or normal cell toxicity activity. By applying this SAR, researchers can achieve high-efficiency, low-toxicity anti-tumor DLCs through rational design. More importantly, our study discovered the compound **5BMF** that displays significant anti-tumor activity not only in 10 cancer cell lines but also in nude mice implanted with human NSCLC. In addition to cancer treatment, **5BMF** can also be used as a fluorescence imaging probe for tumor imaging. Thus, F16s have a bright future for use as a platform for developing targeted theranostic agents.

## Conflicts of interest

There are no conflicts to declare.

## Supplementary Material

Supplementary information
