# Supplementary material for: Mitochondria-targeting fluorescent molecules for high efficiency cancer growth inhibition and imaging
Source: Chem Sci. 2019 Jul 8;10(34):7946–51. doi: 10.1039/c9sc01410a (PMC6836573; doi:10.1039/c9sc01410a)
Supplement: Supplementary file 1 [file SC-010-C9SC01410A-s001.pdf]

Electronic Supplementary Material (ESI) for Chemical Science.
This journal is © The Royal Society of Chemistry 2019

Electronic Supplementary Information

Mitochondria-targeted fluorescent molecules for high efficiency cancer growth inhibition and imaging

Hao Chen,‡[a, c] Jing Wang,‡[a, b] Xin Feng,[b, d] Mark Zhu,[b] Simon Hoffmann,[b] Alex Hsu,[b] Kun Qian,[b] Daijuan Huang,[b] Feng Zhao,[b] Wei Liu,[a] Huimao Zhang*[a] and Zhen Cheng*[b]

[a] Department of Radiology, The First Hospital of Jilin University, Changchun, 130021, China

[b] Molecular Imaging Program at Stanford (MIPS), Bio-X Program, and Department of Radiology, Canary Center at Stanford for Cancer Early Detection, Stanford University, California, 94305-5344, USA

[c] Center for Molecular Imaging Research, Shanghai Institute of Materia Medica, Chinese Academy of Sciences, Shanghai, 201203, China

[d] The College of Veterinary Medicine, Jilin University, Changchun, 130021, China

‡ These authors contributed equally to this work.

*** Correspondence should be sent to: zcheng@stanford.edu, huimaozhanglinda@163.com**

DOI: 10.1039/x0xx00000x

Supplementary figures

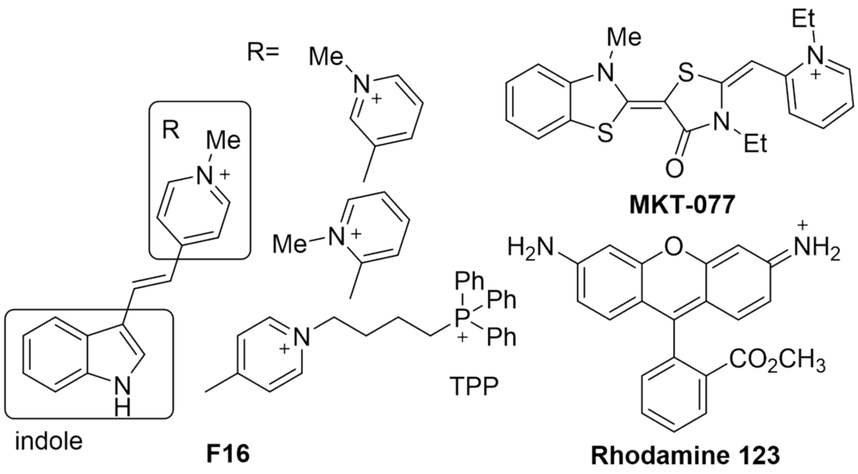

Figure S1. Structure of MKT-077, Rhodamine 123, F16 and its' derivatives

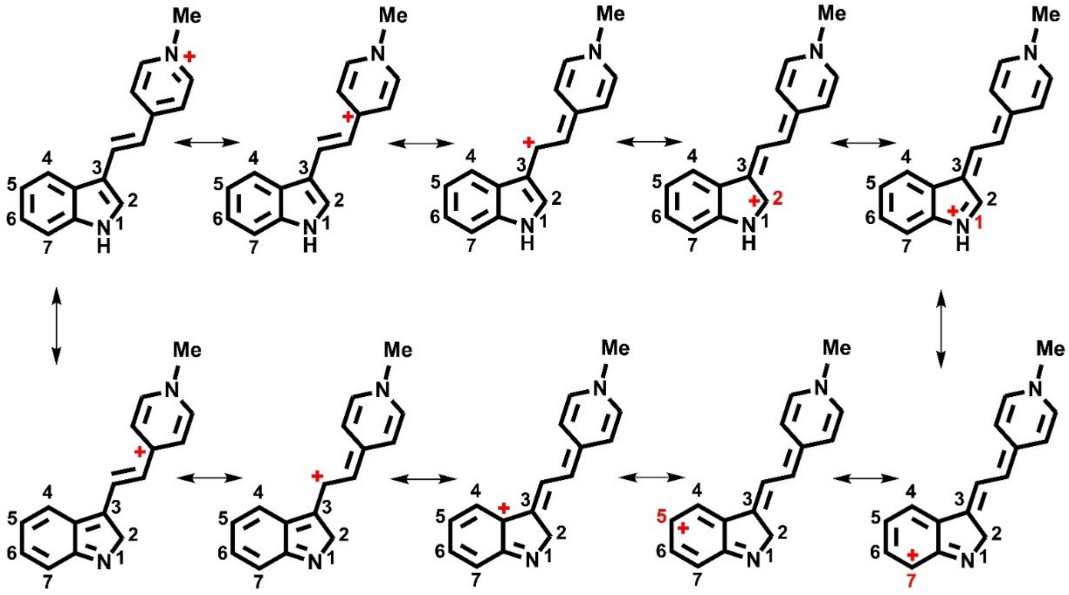

Figure S2. F16 tautomerization analysis. Positive charge priority spreads at 1, 2, 5, 7 positions.

1. 1-MeFF16

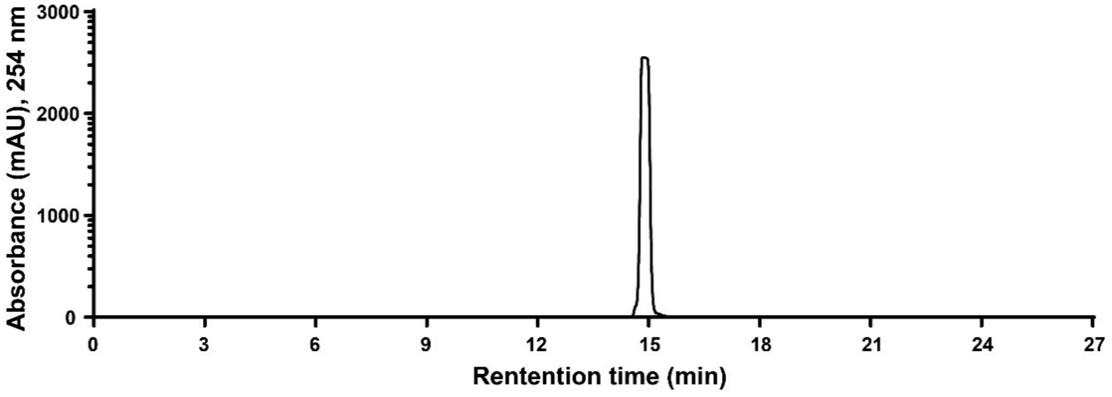

2. PhF16

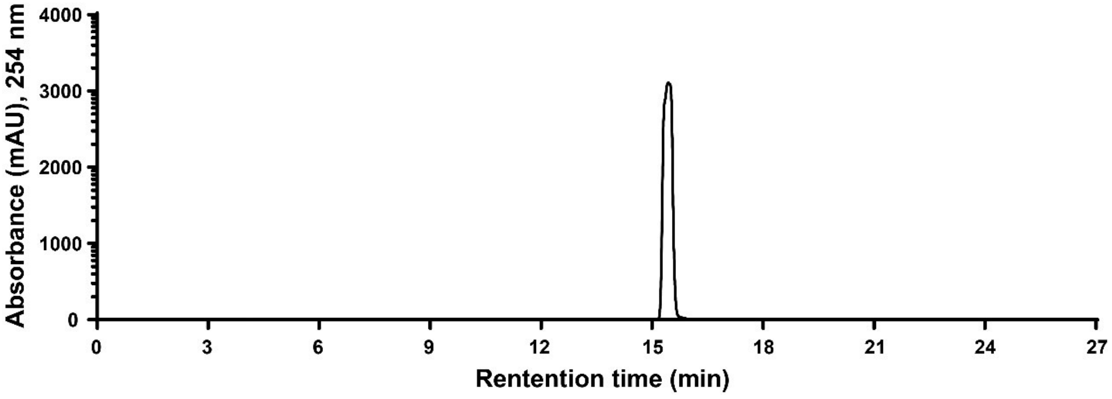

3. 5-Br-7-MeF16
(5BMF)

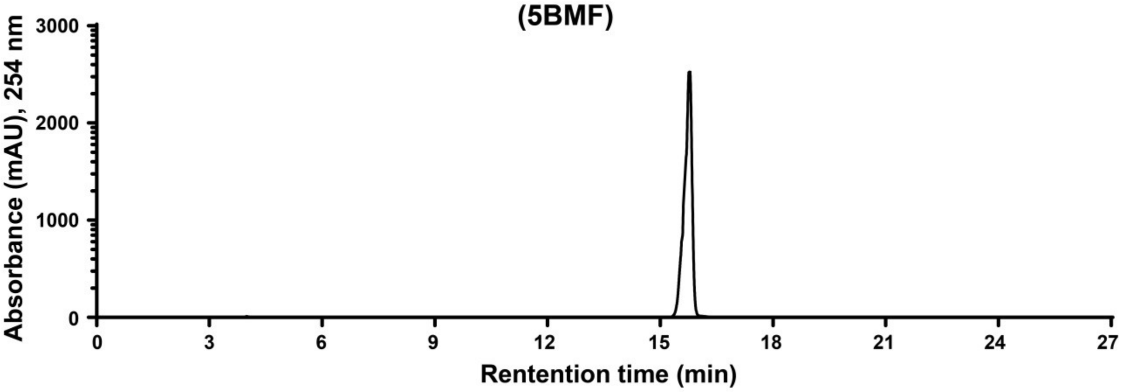

4. 5-BrF16

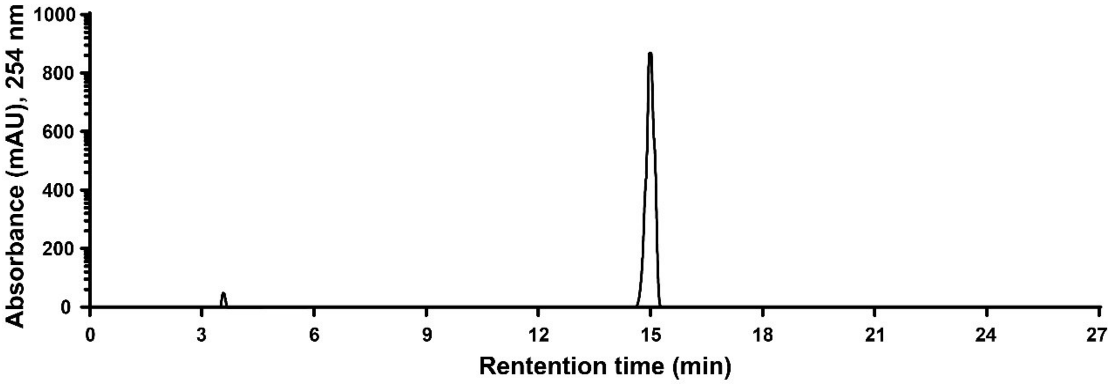

5. 2-PhF16

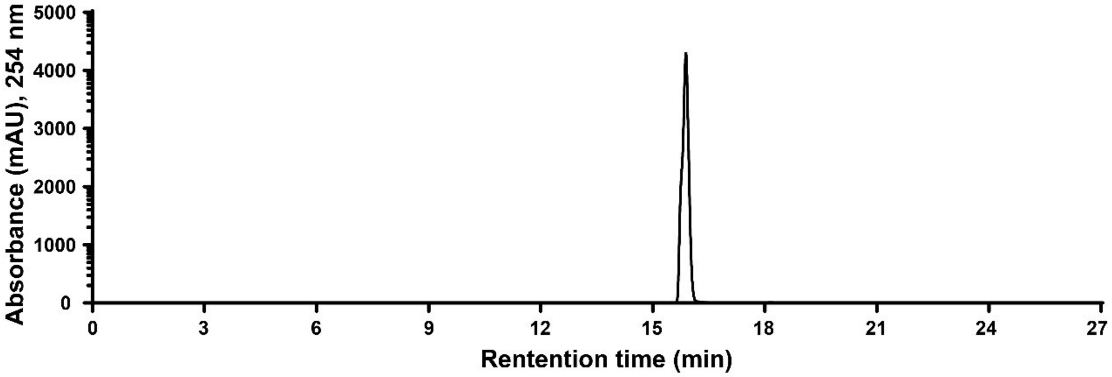

6. 5-CNF16

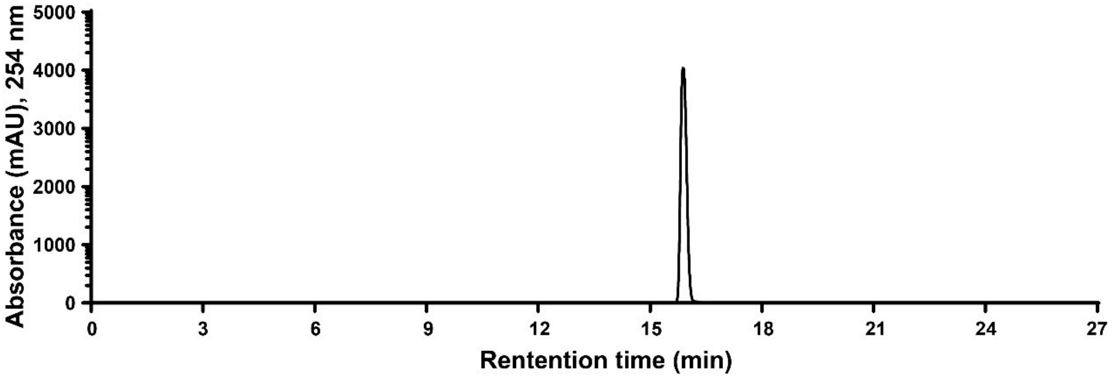

7. FF16

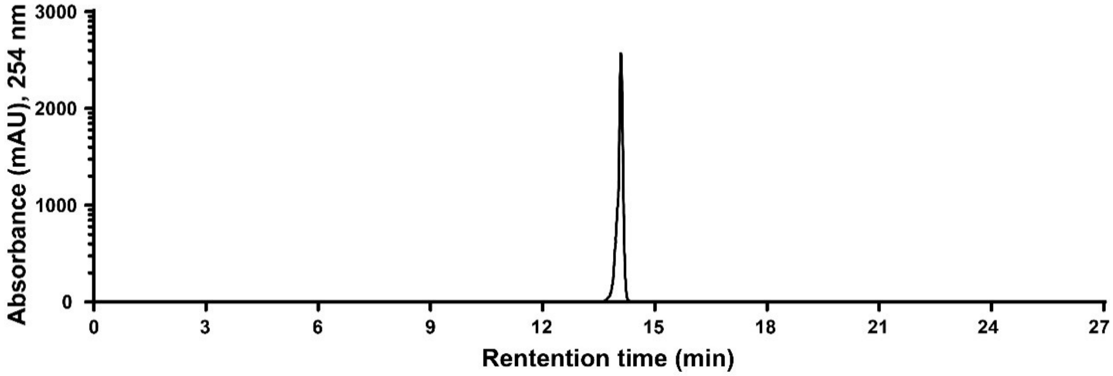

8. 2-ClF16

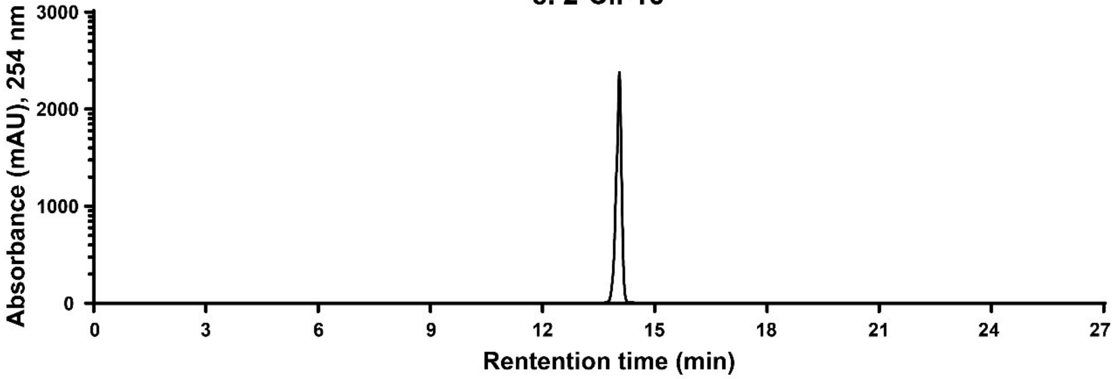

9. 2-Me-5-MeF16

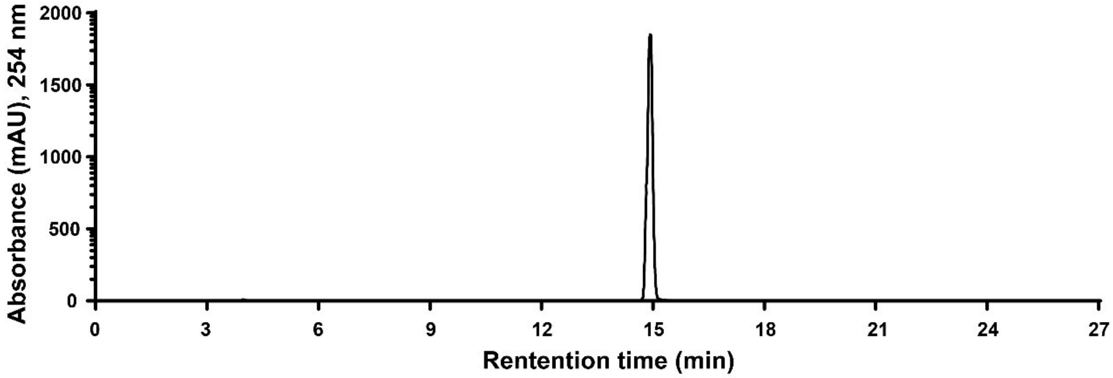

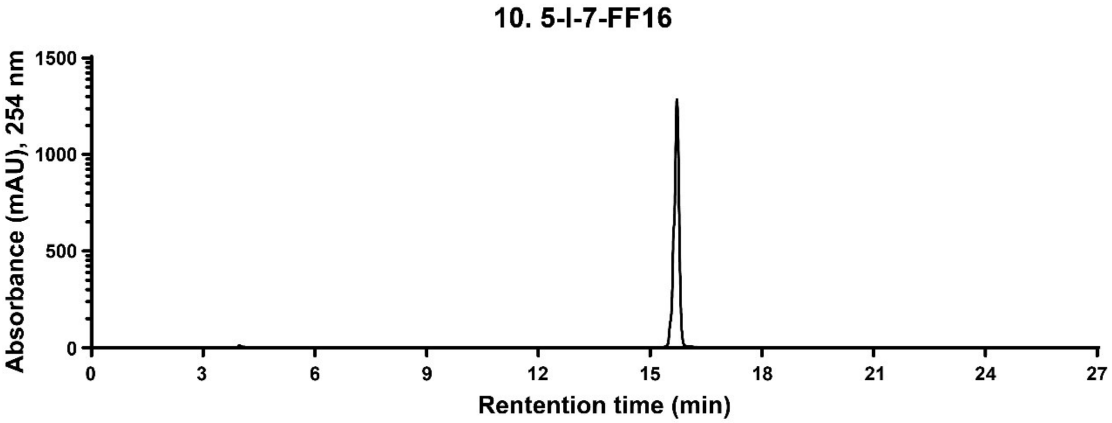

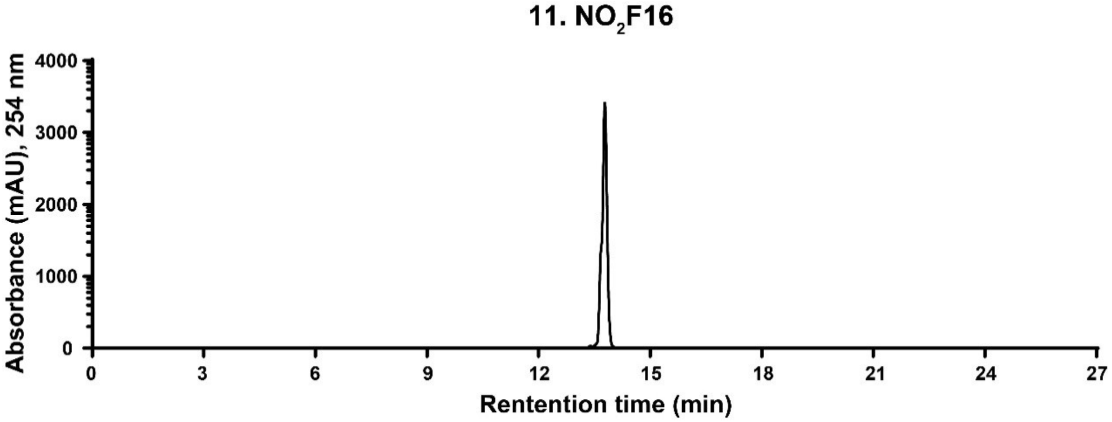

Figure S3. Analytical high-performance liquid chromatography (HPLC) chromatograms. The purity of F16s was detected by analytic HPLC, a Dionex Summit HPLC system (Dionex Corporation, Sunnyvale, CA) with a 340U four-channel UV-Vis absorbance detector, reverse-phase Dionex Acclaim 120 (C18, 4.6 mm × 250 mm) analytic column was used. The mobile phase was water and acetonitrile (both containing 0.1% TFA). The flow rate was 1mL/min with gradient elution starting from 5% acetonitrile (0.1% TFA) and ending up with 95% acetonitrile (0.1% TFA) at 27 mins. 254 nm was used as the detection wavelength. The final purity of all F16s is above 98%.

Compound	Maximum solubility in water at room temperature (mM)
1. 1-MeFF16	1.121
2. PhF16	0.962
3. 5BMF	1.27
4. 5-BrF16	1.536
5. 2-PhF16	0.861
6. 5-CNF16	1.502
7. FF16	1.108
8. 2-ClF16	1.241
9. 2-Me-5-MeF16	1.153
10. 5-I-7-FF16	0.867
11. NO_2F16	1.103

Figure S4 F16s' maximum solubility in water at room temperature.

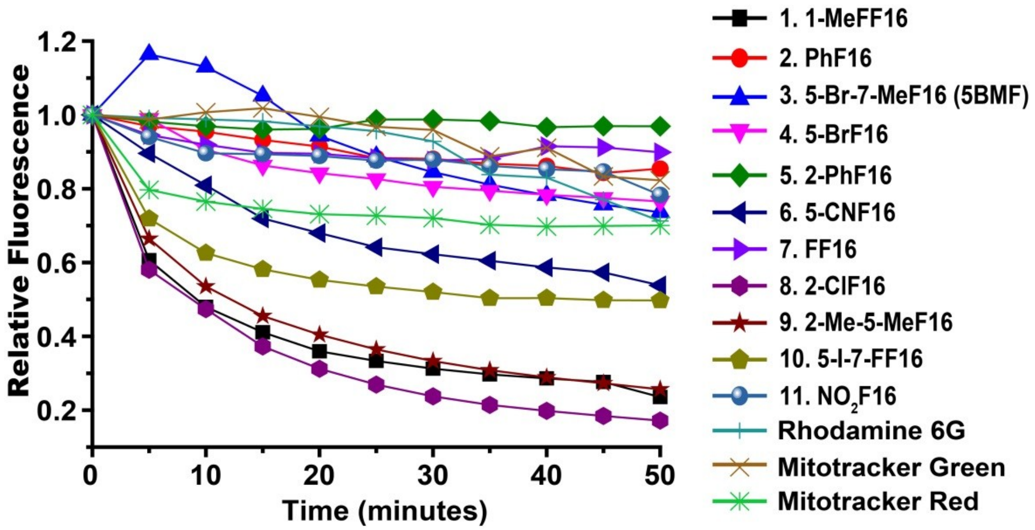

Figure S5. Photostability of F16s (measured in water 10 μM)

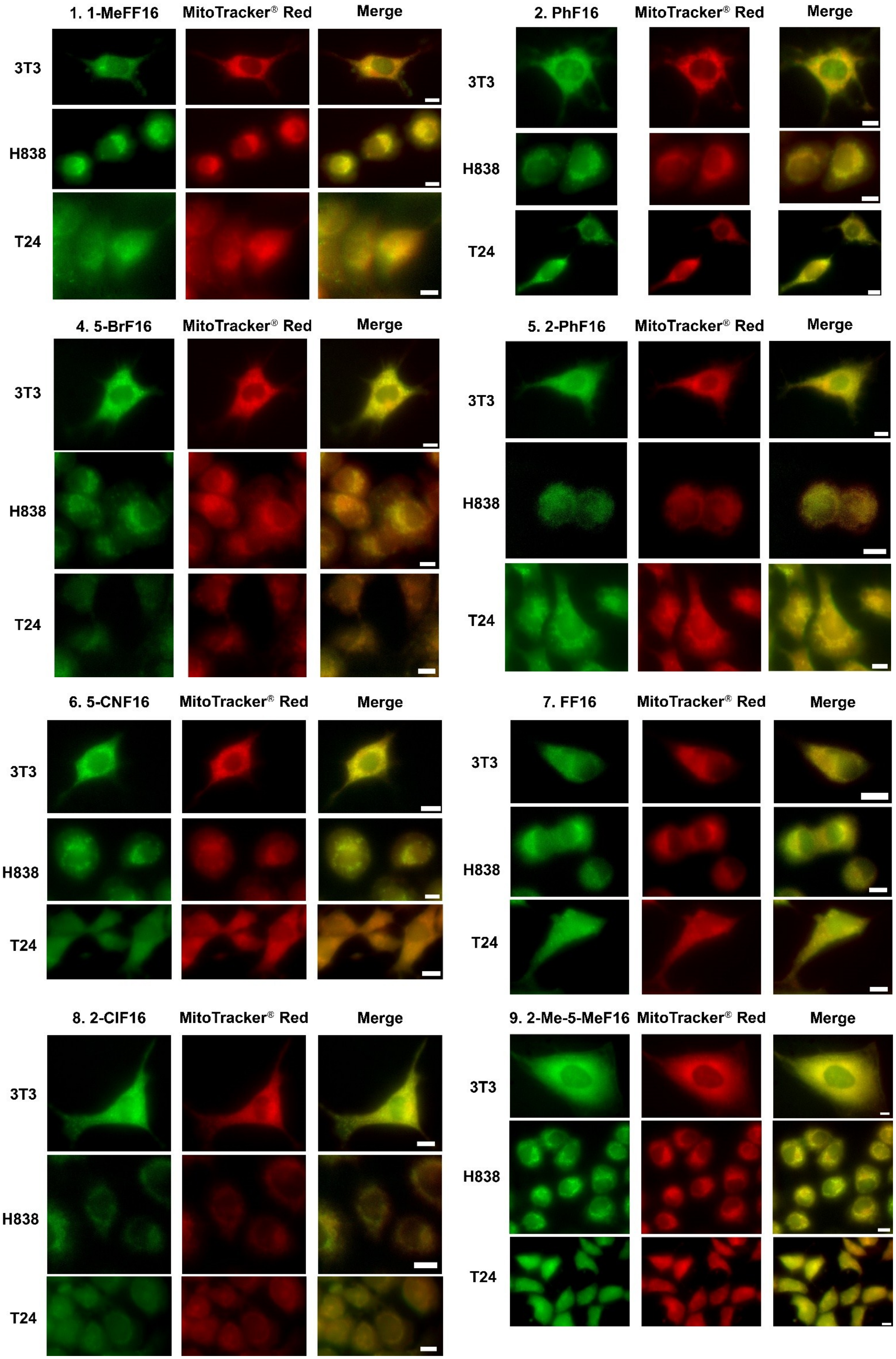
1. 1-MeFF16
MitoTracker® Red
Merge
3T3
H838
T24
2. PhF16
MitoTracker® Red
Merge
3T3
H838
T24
4. 5-BrF16
MitoTracker® Red
Merge
3T3
H838
T24
5. 2-PhF16
MitoTracker® Red
Merge
3T3
H838
T24
6. 5-CNF16
MitoTracker® Red
Merge
3T3
H838
T24
7. FF16
MitoTracker® Red
Merge
3T3
H838
T24
8. 2-ClF16
MitoTracker® Red
Merge
3T3
H838
T24
9. 2-Me-5-MeF16
MitoTracker® Red
Merge
3T3
H838
T24

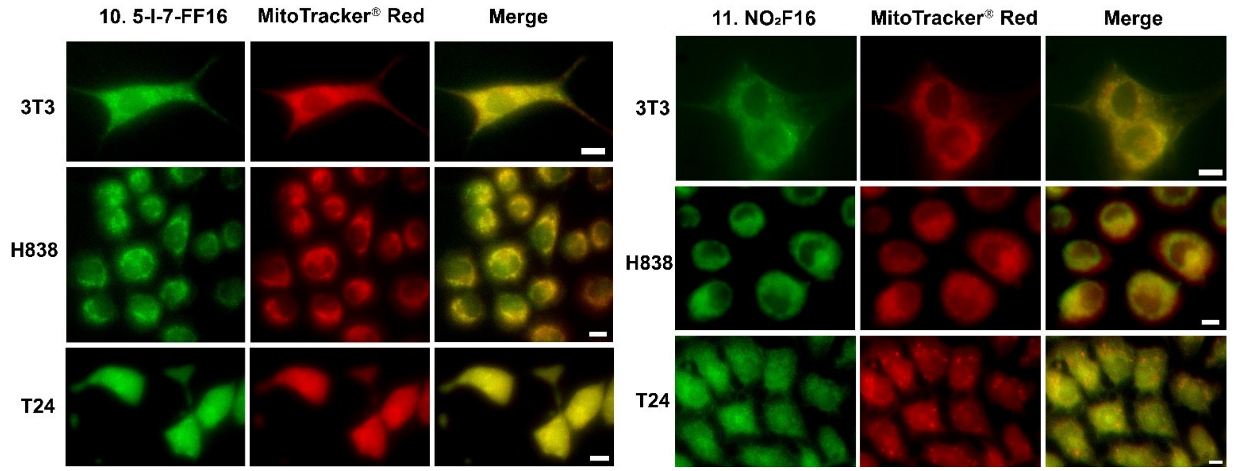

Figure S6. Colocalization of **F16s** (GFP channel) with mitochondrial specific probe MitoTracker® red (microscopy RFP channel), 63 × oil objective lens. White scale bar: 10 μm

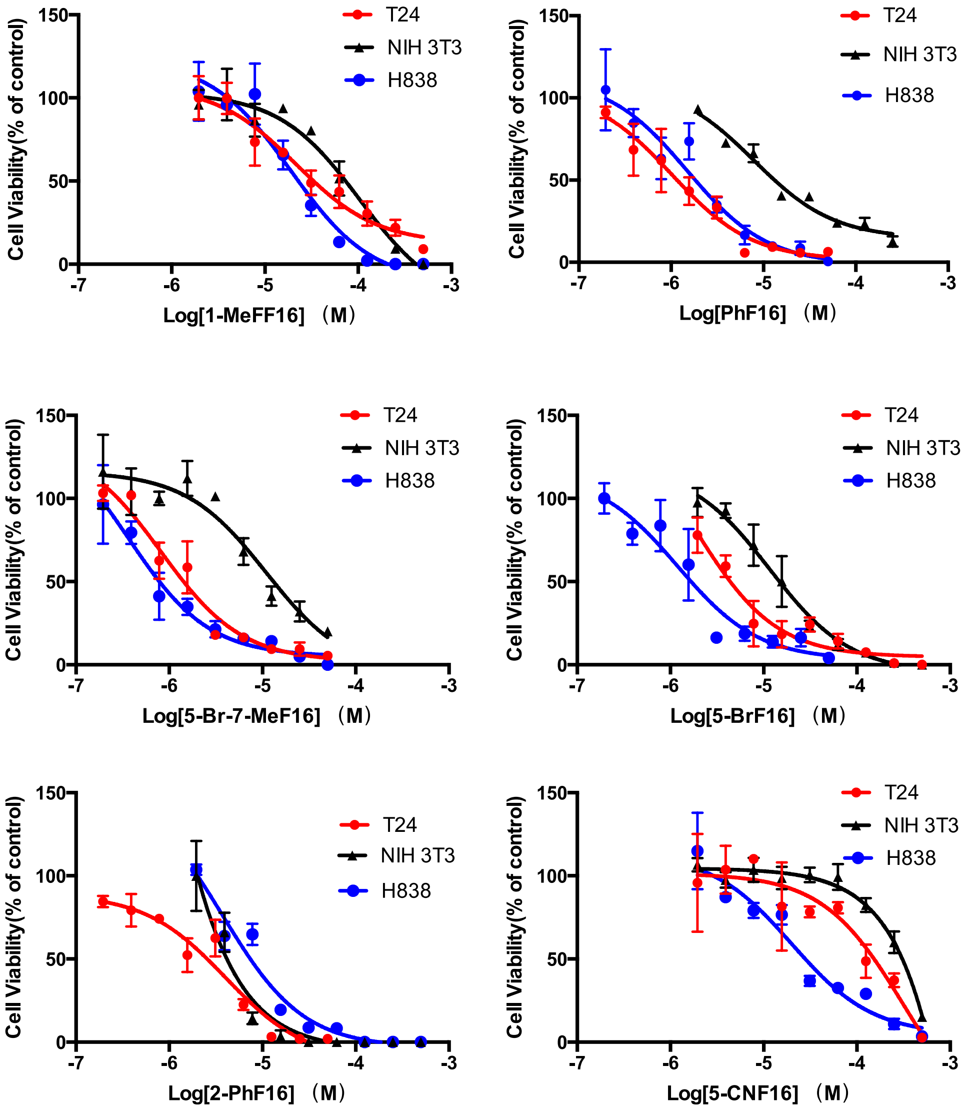

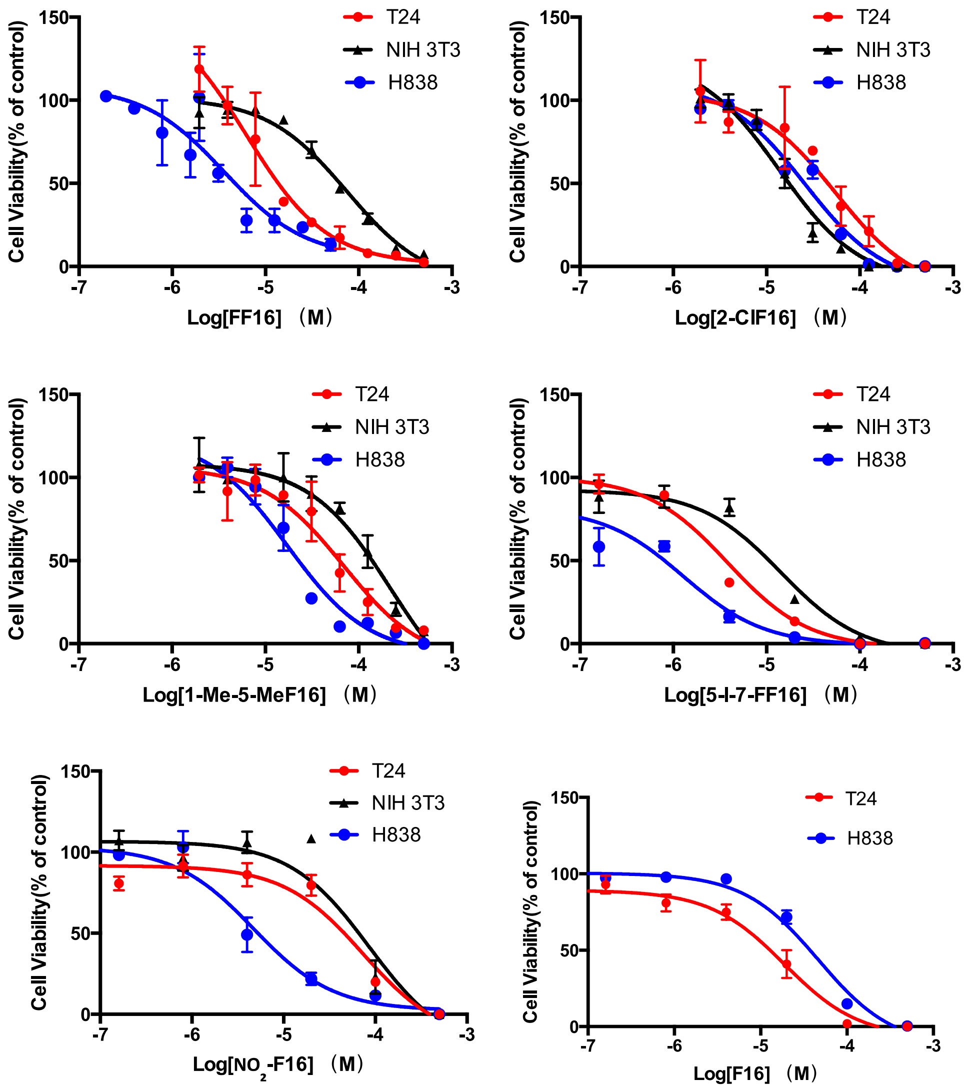

Cell Viability(% of control)
150
100
50
0
-7
-6
-5
-4
-3
T24
NIH 3T3
H838
Log[FF16] (M)
Log[2-ClF16] (M)
Log[1-Me-5-MeF16] (M)
Log[5-I-7-FF16] (M)
Log[NO2-F16] (M)
Log[F16] (M)

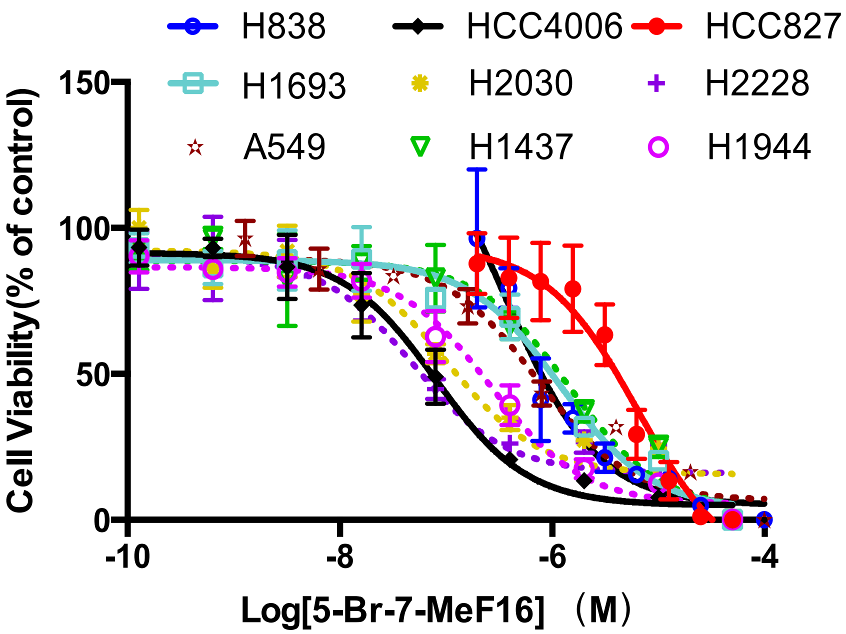

Figure S7. Antiproliferative effect of F16s. Data are expressed in cell proliferative ratio with exposure of the F16s for 4 days to the negative control in PBS buffer. The proliferation status of treated cultures was determined by direct counting of cells. Data are presented as mean ± SD (n=6).

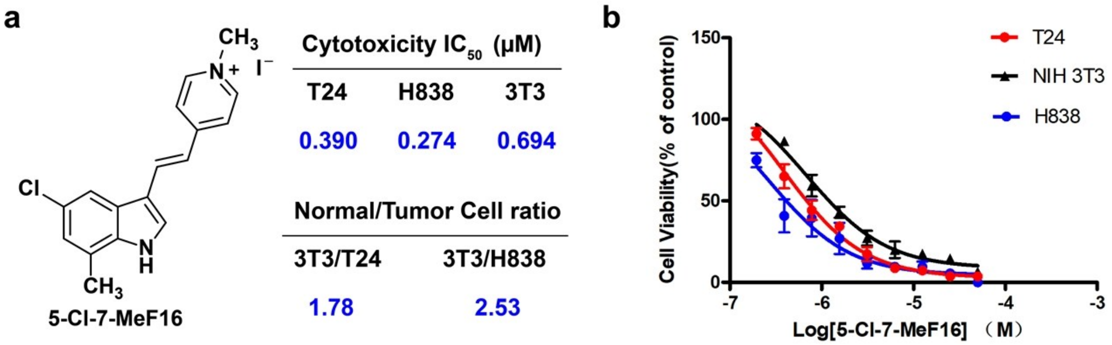

Figure S8. **a**, the chemical structure of **5-Cl-7-MeF16**(left), as well as it's T24, H838, 3T3 cytotoxicity IC_{50} and normal cell to tumor cell ratio(right); **b**, antiproliferative effect curve of **5-Cl-7-MeF16**

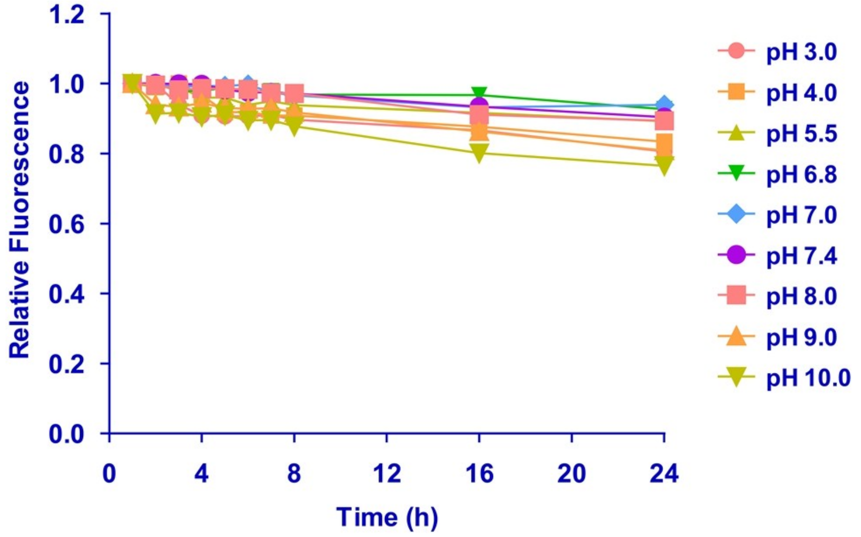

Figure S9. pH stability of **5BMF** (measured in pH 3.0, 4.0, 5.5, 6.8, 7.0, 7.4, 8.0, 9.0, 10.0 buffer with 10 μM concentration). pH 3.0~7.0 buffers are sodium hydrogen phosphate and trisodium buffer; pH 7.4~10.0 buffers are phosphate buffered saline. **5BMF** was put into different pH buffer with the concentration of 10 μM. **5BMF** solutions were taking out at 1, 2, 3, 4, 5, 6, 7, 8, 16, 24 h time point. And their integrated fluorescent intensity (from 450 nm to 650 nm) were measured (excited at 425 nm). All data were analyzed using Origin Pro 9.0 software (OriginLab, Northampton, Massachusetts, USA).

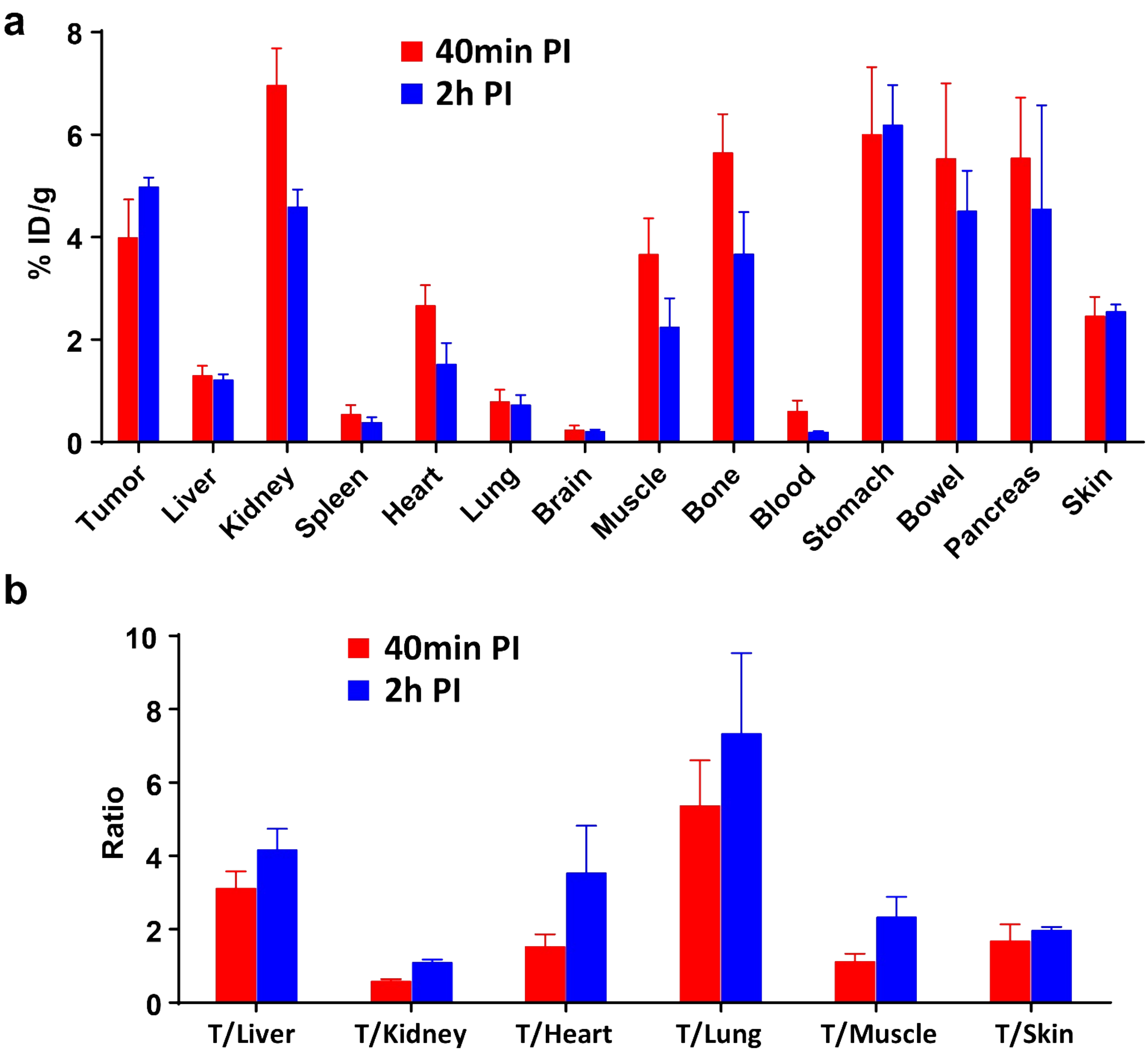

Figure S10. Fluorescent intensity (**a**) and tumor to normal tissue ratio (**b**) of different organs. (n=3 per group). Red: 40 min post-injection (PI); Blue: 2 h PI.

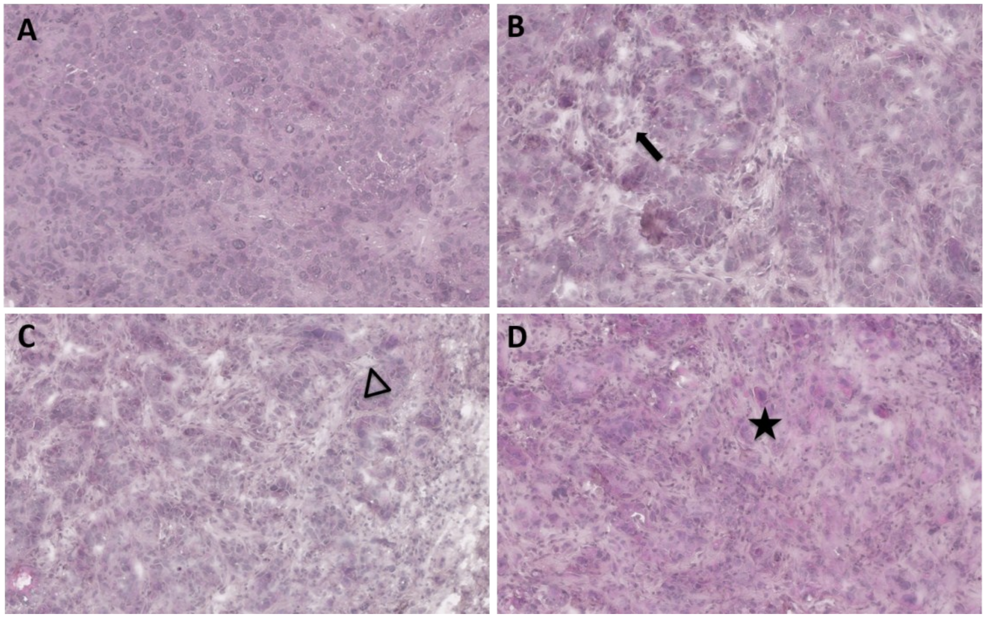

Figure S11. HCC827 tumor pathological section H&E staining (×20). **A**. Untreated tumor tissue; **B-D**. **5BMF**-treated tumor tissues H&E staining of frozen tumor tissue sections showed **5BMF** caused tumor cell density decreased, inflammatory cells infiltrated (🡔), fibrous tissue proliferated (Δ) and cell apoptosis (★). **5BMF** seems to cause tumor inflammatory cell infiltration, fibrous tissue proliferation, and cell apoptosis.

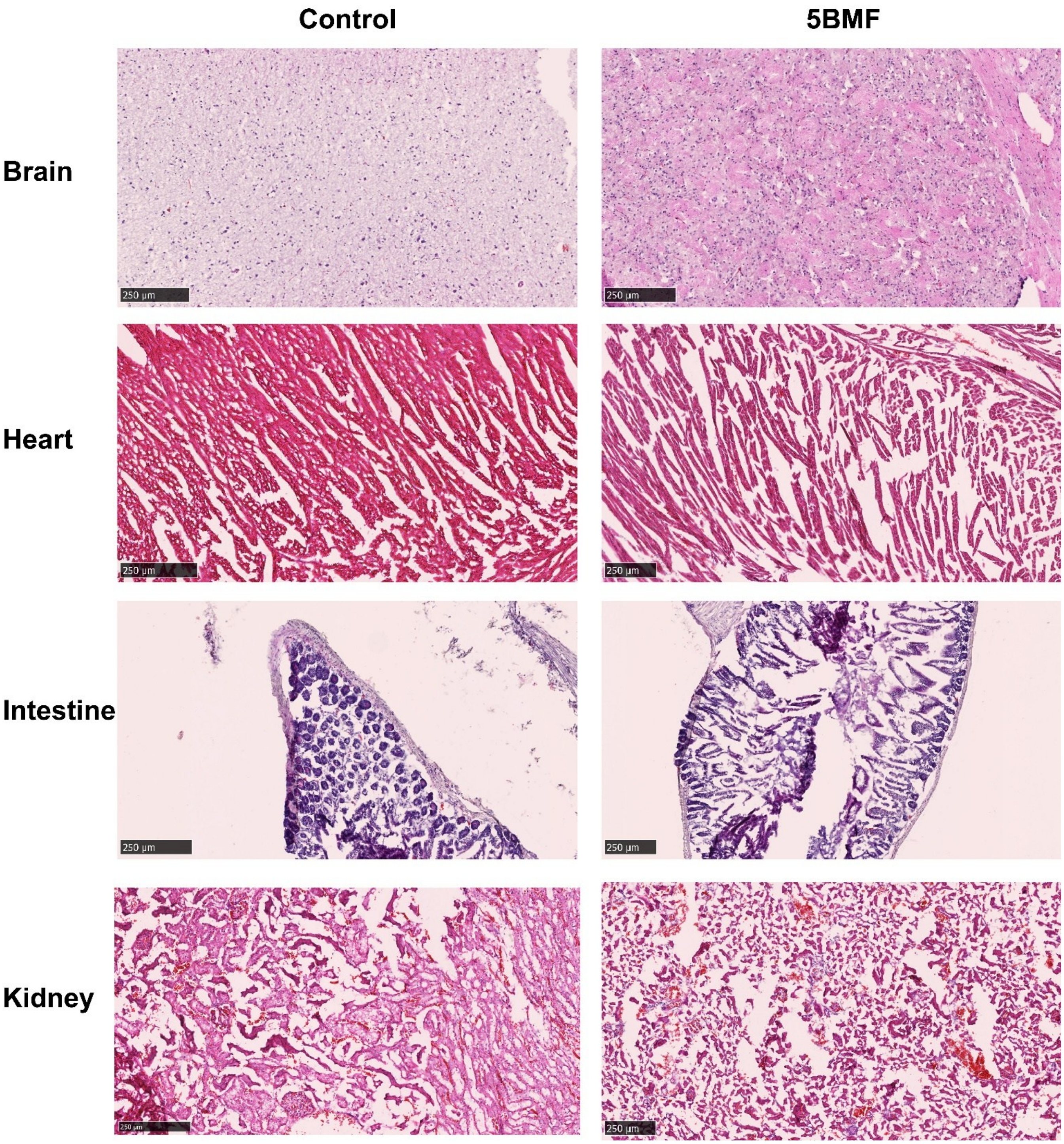
Control
5BMF
Brain
Heart
Intestine
Kidney
250 µm

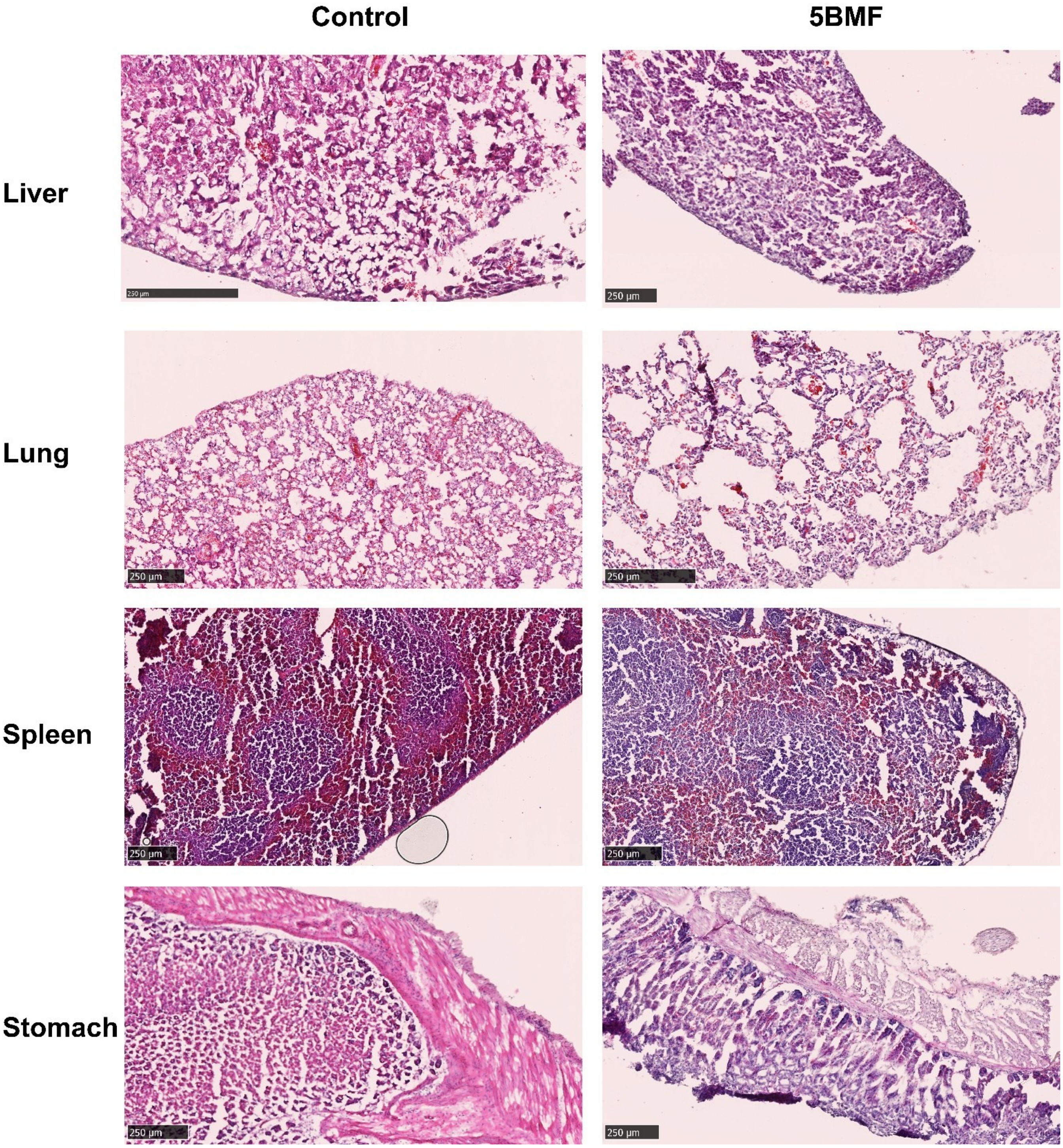

Figure S12. Histologic and microscopic examinations of major organs of HCC827 tumor-bearing mice after the antitumor treatment with either PBS buffer or 5BMF (IV injection of 15 mg/Kg per mouse, injections were given on day 1, 3, 5, 7, 9, 11, 13, 15, 17, 19, 21.). Major organs were stained with hematoxylin and eosin (H&E). Histologic examination of the major organs (including brain, heart, intestine, kidney, liver, lung, spleen, stomach) did not reveal any significant

microscopic lesions in treated mouse group (n = 3) over three weeks, compared to the control group. Black scale bar represents 250 µm.

Experimental Procedures

General Information

All air and moisture sensitive reactions were carried out in flame-dried glassware under a nitrogen atmosphere. Reactive liquid compounds were measured and transferred by gas-tight syringes and were added in the reaction flask through rubber septa. Tetrahydrofuran (THF) was freshly distilled from sodium benzophenoneketyl. Dichloromethane, toluene, and DMF were distilled from CaH_2. All standard synthesis reagents were purchased from Sigma-Aldrich Chemical Co. (St. Louis, MO) and used without further purification. The cell line was obtained from the American Type Tissue Culture Collection (Manassas, VA). Female athymic nude mice (nu/nu) were purchased from Charles River Laboratories (Boston, MA). Analytical thin layer chromatography was performed on glass-backed silica gel plates with F254 indicator. Compounds were visualized under UV lamp or by developing in iodine, vanillin, phosphomolybdic acid solution or with a potassium permanganate solution followed by heating on a hot plate to approximately 350 °C. Flash chromatography was performed on 230-400 mesh silica gel with technical grade solvents which were distilled prior to use. ^{1}H NMR spectra were recorded on a Bruker AV400 at 400 MHz as $CDCl_3$ solutions with tetramethylsilane (δ = 0 ppm) as the internal standard. ^{13}C spectra were obtained on the same instruments at 100 MHz with $CDCl_3$ (δ = 77 ppm) as the internal reference. Chemical shifts are reported in parts per million (ppm). Multiplicities are reported as s (singlet), d (doublet), t (triplet), q (quartet), m (multiplet), dd (doublet of doublet), etc. High-resolution mass spectra were performed on Bruker APEX III 7.0 Tesla Ion Spec 4.7 Tesla FTMS and Thermo Scientific LTQ ORBITRAP XL. Matrix-assisted laser desorption/ionization time of flight mass spectrometry (MALDI-TOF-MS) by Stanford Protein and Nucleic Acid Biotechnology Facility, Stanford University. Analytical or preparative high performance liquid chromatography (HPLC) was performed on a DIONEX ultimate 3000 instrument with PDA detection.

F16 derivatives synthesis

F16 derivatives were synthesized using the reported F16 synthesis method[1]. 1, 4-dimethylpyridium iodide (1.1 mmol) and the different indole-3-carboxaldehyde derivatives (1 mmol) were added into to 10 mL of methanol. After stirring for ten minutes, catalytic amounts of piperidine (0.2 mmol) were added into the mixture. Under nitrogen protection, the reaction solutions were heated to reflux and kept at reflux for 4 to 24 hours, depending on the derivative. The reaction solution changed from light yellow to dark brown, with brown precipitates also forming. Thin layer chromatography analysis was used to detect the reaction endpoint. After the indole-3-carboxaldehyde derivatives disappeared, the reactions were stopped. The products were purified either through using ether recrystallization and semi-preparative HPLC. For recrystallization, the corresponding precipitates were collected, washed with methanol and recrystallized in acetonitrile, giving an orange or brown powder as the product. For the semi-preparative HPLC purification, a Dionex Summit high-performance liquid chromatography (HPLC) system (Dionex Corporation, Sunnyvale, CA) with a 340U four-channel UV-Vis absorbance detector, reverse-phase semi-preparative HPLC column Zorbax SB (C18, 9.4 mm × 250 mm) was used. The mobile phase was water and acetonitrile (both containing 0.1% TFA). The flow was 3mL/min with gradient elution starting from 5% acetonitrile and ending up with 95% acetonitrile at 27 mins. 254 nm and 650 nm were used as the detection wavelength. The purity of F16 products was detected by analysis HPLC with Dionex Acclaim 120 (C18, 4.6 mm × 250 mm) analysis column, 1mL/min flow rate, running the same gradient. All products are above 98% purity. All NMR spectra (^{1}H, ^{13}C) were performed on a Varian XL-400 (Varian, Palo Alto, CA). Electron spray ionization (ESI) mass spectrometry was performed by Vincent Coats Foundation Mass Spectrometry Laboratory, Stanford University.

F16 derivatives optical properties

Absorbance and fluorescence. The UV absorbance of F16s was recorded on an Agilent 8453 UV spectrophotometer at a concentration of 31 μM in water. Fluorescence was recorded on a Fluoromax-3 spectrofluorometer (Jobin Yvon) at a concentration of 7.8 μM in water.

Photostability. A 75 W Xenon arc lamp (Hamamatsu, San Jose, California, USA) with 420-470 nm bandpass filter (MF445-45, Thorlabs, Newton, New Jersey, USA) was used as the light source for the F16 derivatives, Rhodamine 6G, and Mitotracker Green. Analogously, a 540-580 nm

bandpass filter (MF559-34, Thorlabs, Newton, New Jersey, USA) was used to excite Mitotracker Red. All the dyes were dissolved in water at a 10 μM concentration in 700 μL micro quartz cuvettes (10 mm, Sigma-Aldrich, St. Louis, MO, USA). They were continuously excited for 50 mins. During this period, the fluorescence intensity was measured every 5 mins at 525 nm (F16s, Rhodamine 6G, Mitotracker Green) or 600 nm (Mitotracker Red). Relative fluorescence intensity change was monitored over time.

Quantum yield. The fluorescence quantum yields of F16s were determined according to the literature[2].

$$\Phi x=\Phi s(Fx/Fs)\ (As/Ax)$$

Where Φ is the quantum yield, F is the integrated area under the corrected emission spectrum, and A is the absorbance at the excitation wavelength; the subscripts x and s refer to F16s and the standard, respectively. Rhodamine 6G (ΦF = 95%) in ethanol was used as the standard. All F16s and Rhodamine 6G were dissolved in ethanol in 5 different concentrations with the absorbance lower than 0.1 at 425nm. The corresponding 5 different fluorescences of F16s and Rhodamine 6G were excited at 425 nm and the emission spectra from 450 nm to 650 nm were obtained for the following integrated area measurement. All data were analyzed using Origin Pro 9.0 software (OriginLab, Northampton, Massachusetts, USA) to get the F16s final quantum yield.

Cell culture, imaging, and cytotoxicity assay

Cell culture. H838, HCC4006, HCC827, H1693, H2030, H2228, H1437, H1944 were cultured in RPMI-1640 medium. NIH/3T3 was cultured in DMEM medium. A549 was cultured in F-12K medium. T24 were cultured in McCoy's 5A medium. All culture mediums were supplemented with 10% fetal bovine serum. The cells were incubated at 37 ^{o}C in an atmosphere containing 5% CO_2.

Cell Imaging. For the F16 derivatives' live cell localization imaging, 3 μM F16s compounds were added to cells grown in a MatTek glass-bottom culture dishes (Ashland, Massachusetts) for 1 h and washed with PBS (phosphate-buffered saline) three times. After replacement of the medium, cells were imaged using a fluorescent microscope (Zeiss) with a 63× oil or 20× objective lens (excited in the GFP channel).

Mitotracker® Red (Molecular Probes) and Hoechst (Thermo Scientific) were added to the medium to stain the mitochondria and nuclei, following the manufacturer's procedures. Cells were photographed as previously described using a 63× oil or 20× objective lens.

The effect of membrane potential on **5BMF** accumulation was performed through staining HCC827 cells in low K^+ and high K^+ buffer, instead of RPMI-1640 culture medium. Low K^+ buffer contains 137 mM NaCl, 3.6 mM KCI, 0.5 mM $MgCl_2$, 1.8 mM $CaCl_2$ of RPMI-1640 medium (GIBCO). High K^+ buffer was prepared the same as low K^+ buffer, with the exception of 137 mM KCl and 3.6 mM NaCl instead. HCC827 cells were incubated in 5 ml low K^+ or high K^+ buffer, containing **5BMF** (3 μM) for 30 mins, then washed 3 times with 5 mL in the same buffer. The buffer was maintained for fluorescent microscope imaging (Zeiss, 20× objective lens, GFP channel).

The effect of the protonophore FCCP (Sigma) on **5BMF** accumulation was performed the same as previously done for F16s localization staining. HCC827 cells preincubated with 5BMF (3 μM) were subsequently treated with 5 μM FCCP. Images were acquired immediately before addition, then 1 minute and 3 minutes after FCCP addition.

Cytotoxicity assay. All test cell lines were incubated in 96-well plates overnight at a density of 3000 cells/well. The culture medium was replaced with 200 μL of culture medium, into which the testing compound was dispersed at various concentrations (1.95 - 500 μM). After incubating for 3 days, the viable cell numbers were checked and directly counted under microscopy (10X). A minimum of 1mm×1mm area was counted from each of at least three widely separated regions of cell culture. The cell proliferation rate was calculated by the following formula: cell proliferation rate (%) = (average cell number of sample wells / average cell number of control wells) ×100%. The intact culture medium was evaluated as a control.

HCC827 s.c. tumor model treatment and imaging

All animal experiments were performed in compliance with the Guidelines for the Care and Use of Research Animals as established by the Stanford University Animal Studies Committee. Female athymic nude mice (nu/nu) from 4−6 weeks old were obtained from Charles River Laboratories (Boston, MA, USA) and kept under sterile conditions. 5×10^6 human lung adenocarcinoma

HCC827 cells suspended in 150 μL of PBS were inoculated subcutaneously in the right shoulder of the nude mice.

When the tumors reached ~3 mm in diameter, the tumor-bearing mice were administered i.v. with **5BMF** (dissolved in 150 μL PBS, with 2 μL DMSO) at 15 mg/Kg on days 1, 3, 5, 7, 9, 11, 13, 15, 17, 19, 21. Correspondingly, untreated tumor-bearing mice serving as controls were administered i.v. with 150 μL 1× PBS pH 7.4 (with 2 μL DMSO) on the same days as the treated mice. Ten mice were used for each group. Length and width of the elliptical tumors were measured by Vernier caliper after every intravenous injection. Modified ellipsoid formula ½ (Length × $Width^2$) was used to calculate the tumor size. The tumor growth was calculated with the ratio of final volume (FV) minus initial volume (IV) to initial volume (IV).

In vivo fluorescent imaging was taken 40 minutes and 2 hours after the injection of **5BMF** 15 mg/kg. IVIS spectrum instrument was used. The excitation wavelength was set at 450 nm. Collection wavelength was set at 550 nm, using a 3s acquisition time. 4 mice for each group were used. Mice were sacrificed and the organs were harvest after each set of in vivo imaging. Fluorescent images of the organs were taken under the same settings.

Histological study

The tumor tissues and some important organs such as liver, kidney, lung, heart were isolated. After frozen, the specimens sectioned at 5-8 μm and processed for a standard hematoxylin-eosin (H&E) staining. Slides were viewed and photographed with the Nanozoomer Digital Pathology Image (NDPI).

Statistical analysis

The body weights of mice are presented as means ± standard deviation (SD) of n independent measurements. The tumor volumes are presented as means ± standard error of mean (SEM) of n independent measurements. Statistical analysis was performed using a Student's t-test or Mann-Whitney U test. Statistical significance was assigned for P values <0.05.

Detail F16s Synthesis information

1. Synthesis of compound **1, 1-MeFF16**

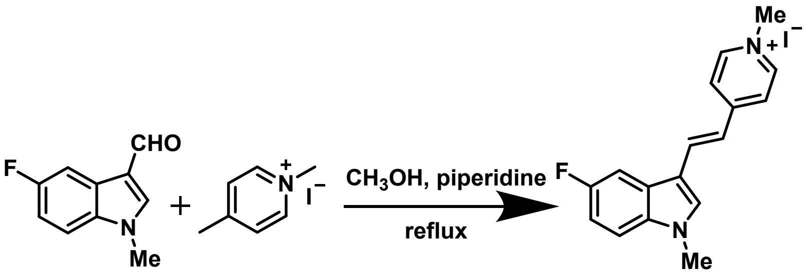

Put 258.6 mg 1,4-dimethylpyridium iodide (1.1 mmol) and 177.2 mg 5-fluoro-1-methyl-1*H*-indole-3-carbaldehyde (1.0 mmol) into 10 mL methanol. Stirred for 5 mins, then added 19.7 μL piperidine (17 mg, 0.2 mmol). After 10 mins stirring, the reaction mixture was heated to reflux for 6 hours. The color of the reaction change from light yellow to dark brown. Yellow precipitate appeared after 2 hours refluxing. After 6 hours the precipitate was collected, washed with methanol and recrystallized with acetonitrile, then purified with semi-preparative HPLC to give the product as orange powder 315 mg (yield: 80%) ^{1}H NMR (400 MHz, dmso) δ 8.68 (d, J = 6.7 Hz, 2H), 8.19 – 8.10 (m, 4H), 7.94 (s, 1H), 7.46 (dd, J = 10.0, 2.2 Hz, 1H), 7.25 (d, J = 16.3 Hz, 1H), 7.11 (td, J = 9.3, 2.3 Hz, 1H), 4.16 (s, 3H), 3.83 (s, 3H). ^{13}C NMR (101 MHz, dmso) δ 161.14, 158.78, 154.35, 144.69, 138.81, 138.68, 136.39, 135.45, 122.24, 117.74, 113.14, 110.04, 109.80, 98.30, 98.03, 46.78, 33.73. MS(ESI^{+}): 267.1 (for calculated $C_{17}H_{16}FN_2^{+}$ 267.1)

2. Synthesis of compound **2, PhF16**

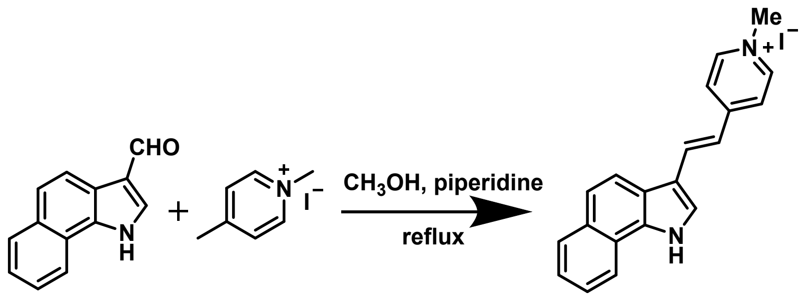

Put 258.6 mg 1,4-dimethylpyridium iodide (1.1 mmol) and 195.2 mg 1*H*-benzo[*g*]indole-3-carbaldehyde (1.0 mmol) into 10 mL methanol. Stirred for 5 mins, then added 19.7 μL piperidine (17mg, 0.2 mmol). After 10 mins stirring, the reaction mixture was heated to reflux overnight. The color of the reaction change from pale brown to red brown. Yellow precipitate appeared after 1

hours refluxing. At the end of the reaction, the precipitate was collected, washed with methanol and recrystallized with acetonitrile, then purified with semi-preparative HPLC to give the product as orange powder 259.6 mg (yield: 63%). ^{1}H NMR (400 MHz, dmso) δ 12.23 (s, 1H), 8.62 (d, *J* = 5.8 Hz, 2H), 8.22 (d, *J* = 7.7 Hz, 1H), 8.04 (d, *J* = 5.7 Hz, 2H), 7.93 (d, *J* = 16.0 Hz, 1H), 7.68 (d, *J* = 6.8 Hz, 2H), 7.62 (t, *J* = 7.0 Hz, 2H), 7.56 (d, *J* = 7.1 Hz, 1H), 7.51 (d, *J* = 7.7 Hz, 1H), 7.42 (d, *J* = 16.1 Hz, 1H), 7.29 (dd, *J* = 16.9, 8.0 Hz, 2H), 4.16 (s, 3H). ^{13}C NMR (101 MHz, dmso) δ 154.32, 144.62, 144.30, 137.39, 135.60, 131.32, 130.06, 129.63, 129.57, 126.06, 123.82, 122.36, 121.97, 121.39, 118.96, 112.75, 110.41, 46.80. MS(ESI$^+$): 285.2 (for calculated $C_{20}H_{17}N_2^+$ 285.1)

3. Synthesis of compound **3, 5BMF**

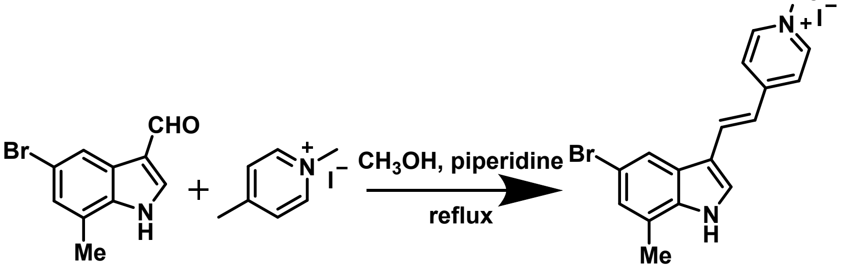

Put 258.6 mg 1,4-dimethylpyridium iodide (1.1 mmol) and 238.1 mg 5-bromo-7-methyl-1H-indole-3-carbaldehyde (1.0 mmol) into 10 mL methanol. Stirred for 5 mins, then added 19.7 µL piperidine (17 mg, 0.2 mmol). After 10 mins stirring, the reaction mixture was heated to reflux overnight. The color of the reaction change from yellow to brown. Yellow precipitate appeared after 1 hours refluxing. After the reaction finished, the precipitate was collected, washed with methanol and recrystallized with acetonitrile, then purified with semi-preparative HPLC to give the product as orange powder 232.1 mg (yield: 51%). ^{1}H NMR (400 MHz, dmso) δ 12.07 (s, 1H), 8.69 (s, 2H), 8.21 (d, J = 16.3 Hz, 1H), 8.12 (d, J = 4.5 Hz, 3H), 8.03 (s, 1H), 7.26 (d, J = 16.3 Hz, 1H), 7.18 (s, 1H), 4.17 (s, 3H), 2.47 (s, 3H). ^{13}C NMR (101 MHz, dmso) δ 154.38, 144.67, 135.97, 135.61, 132.14, 127.02, 126.10, 124.73, 122.27, 120.25, 117.95, 114.37, 113.89, 46.80, 16.85. MS(ESI$^+$): 327.0 (for calculated $C_{17}H_{16}BrN_2^+$ 327.0)

4. Synthesis of compound **4**, **5-BrF16**

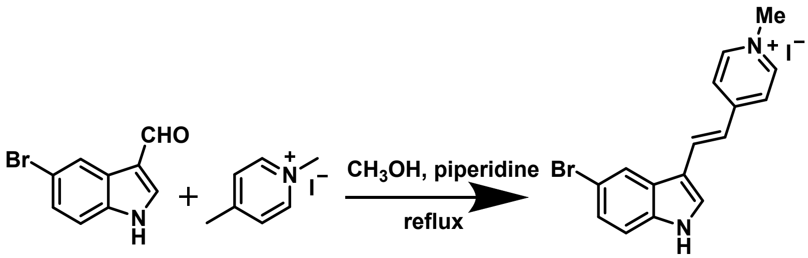

Put 258.6 mg 1,4-dimethylpyridium iodide (1.1 mmol) and 224.1 mg 5-bromo-1*H*-indole-3-carbaldehyde (1.0 mmol) into 10 mL methanol. Stirred for 5 mins, then added 19.7 μL piperidine (17 mg, 0.2 mmol). After 10 mins stirring, the reaction mixture was heated to reflux overnight. The color of the reaction change from yellow to brown. Yellow precipitate appeared after 1 hours refluxing. After the reaction finished, the precipitate was collected, washed with methanol and recrystallized with acetonitrile, then purified with semi-preparative HPLC to give the product as orange powder 242.6 mg (yield: 55%). ^{1}H NMR (400 MHz, dmso) δ 12.04 (s, 1H), 8.70 (d, *J* = 6.3 Hz, 2H), 8.32 (s, 1H), 8.22 (d, *J* = 16.3 Hz, 1H), 8.13 (d, *J* = 6.3 Hz, 2H), 8.03 (s, 1H), 7.45 (d, *J* = 8.6 Hz, 1H), 7.35 (d, *J* = 8.6 Hz, 1H), 7.27 (d, *J* = 16.3 Hz, 1H), 4.17 (s, 3H). ^{13}C NMR (101 MHz, dmso) δ 154.42, 144.70, 136.47, 135.56, 132.79, 127.33, 125.83, 122.79, 122.31, 118.00, 114.92, 114.36, 113.48, 46.80. MS(ESI^{+}): 313.0 (for calculated $C_{16}H_{14}BrN_2^{+}$ 313.0)

5. Synthesis of compound **5**, **2-PhF16**

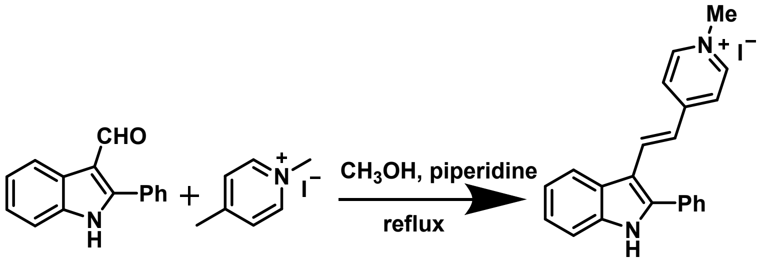

Put 258.6 mg 1,4-dimethylpyridium iodide (1.1 mmol) and 221.2 mg 2-phenyl-1*H*-indole-3-carbaldehyde (1.0 mmol) into 10 mL methanol. Stirred for 5 mins, then added 19.7 μL piperidine (17 mg, 0.2 mmol). After 10 mins stirring, the reaction mixture was heated to reflux overnight. The color of the reaction change from yellow to brown. Yellow precipitate appeared after 1 hours refluxing. After the reaction finished, the precipitate was collected, washed with methanol and recrystallized with acetonitrile, then purified with semi-preparative HPLC to give the product as

orange powder 306.8 mg (yield: 70%). ^{1}H NMR (400 MHz, dmso) δ 12.23 (s, 1H), 8.62 (d, *J* = 5.8 Hz, 2H), 8.22 (d, *J* = 7.7 Hz, 1H), 8.04 (d, *J* = 5.7 Hz, 2H), 7.93 (d, *J* = 16.0 Hz, 1H), 7.68 (d, *J* = 6.8 Hz, 2H), 7.62 (t, *J* = 7.0 Hz, 2H), 7.56 (d, *J* = 7.1 Hz, 1H), 7.51 (d, *J* = 7.7 Hz, 1H), 7.42 (d, *J* = 16.1 Hz, 1H), 7.29 (dd, *J* = 16.9, 8.0 Hz, 2H), 4.16 (s, 3H). ^{13}C NMR (101 MHz, dmso) δ 154.32, 144.62, 144.30, 137.39, 135.60, 131.32, 130.06, 129.63, 129.57, 126.06, 123.82, 122.36, 121.97, 121.39, 118.96, 112.75, 110.41, 46.80. MS(ESI$^+$): 311.2 (for calculated $C_{22}H_{19}N_2^+$ 311.2)

6. Synthesis of compound **6**, **5-CNF16**

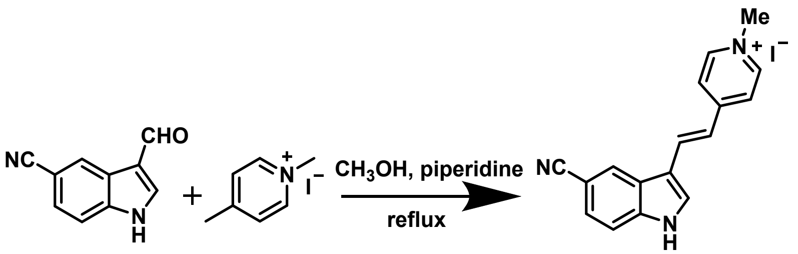

Put 258.6 mg 1,4-dimethylpyridium iodide (1.1 mmol) and 170.2 mg 3-formyl-1H-indole-5-carbonitrile (1.0 mmol) into 10 mL methanol. Stirred for 5 mins, then added 19.7 μL piperidine (17 mg, 0.2 mmol). After 10 mins stirring, the reaction mixture was heated to reflux overnight. The color of the reaction change from yellow to brown. Yellow precipitate appeared after 1 hours refluxing. After the reaction finished, the precipitate was collected, washed with methanol and recrystallized with acetonitrile, then purified with semi-preparative HPLC to give the product as orange powder 240.1 mg (yield: 62%). ^{1}H NMR (400 MHz, dmso) δ 12.29 (s, 1H), 8.74 (d, *J* = 5.5 Hz, 2H), 8.69 (s, 1H), 8.23 (d, *J* = 16.4 Hz, 1H), 8.15 (s, 3H), 7.61 (dd, *J* = 29.8, 8.2 Hz, 2H), 7.38 (d, *J* = 16.3 Hz, 1H), 4.19 (s, 3H). ^{13}C NMR (101 MHz, dmso) δ 154.21, 144.86, 139.51, 134.81, 133.71, 126.10, 125.99, 125.25, 122.52, 120.83, 119.14, 114.35, 114.24, 103.52, 46.95. MS(ESI$^+$): 260.1 (for calculated $C_{17}H_{14}N_3^+$ 260.1)

7. Synthesis of compound **8**, **2-ClF16**

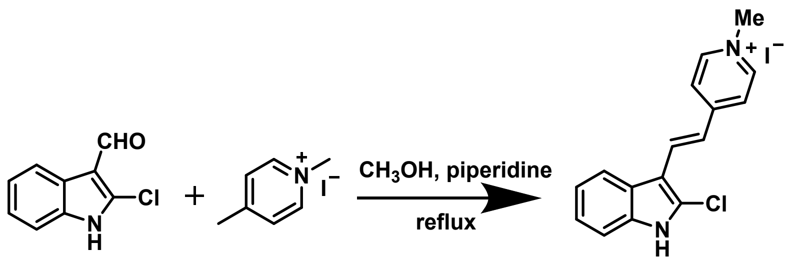

Put 258.6 mg 1,4-dimethylpyridium iodide (1.1 mmol) and 179.6 mg 2-chloro-1*H*-indole-3-carbaldehyde (1.0 mmol) into 10 mL methanol. Stirred for 5 mins, then added 19.7 μL piperidine (17 mg, 0.2 mmol). After 10 mins stirring, the reaction mixture was heated to reflux overnight. The color of the reaction change from yellow to brown. Yellow precipitate appeared after 1 hours refluxing. After the reaction finished, semi-preparative HPLC were used to purify the product. With freeze-dried, the product was gotten as orange powder 178.5 mg (yield: 45%). ^{1}H NMR (400 MHz, dmso) δ 12.89 (s, 1H), 8.71 (d, *J* = 6.5 Hz, 2H), 8.23 (d, *J* = 6.4 Hz, 2H), 8.14 (d, *J* = 7.6 Hz, 1H), 7.95 (d, *J* = 16.3 Hz, 1H), 7.42 (dd, *J* = 11.8, 4.1 Hz, 2H), 7.30 – 7.22 (m, 2H), 4.19 (s, 3H). ^{13}C NMR (101 MHz, dmso) δ 153.94, 144.89, 135.80, 132.25, 129.44, 124.92, 123.81, 122.82, 122.15, 120.32, 119.47, 112.32, 109.38, 46.89. MS(ESI$^+$): 269.1 (for calculated $C_{16}H_{14}ClN_2^+$ 269.1)

8. Synthesis of compound **9**, **2-Me-5-MeF16**

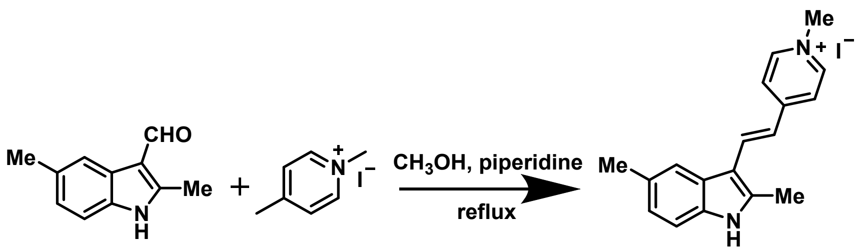

Put 258.6 mg 1,4-dimethylpyridium iodide (1.1 mmol) and 173.2 mg 2-chloro-1*H*-indole-3-carbaldehyde (1.0 mmol) into 10 mL methanol. Stirred for 5 mins, then added 19.7 μL piperidine (17 mg, 0.2 mmol). After 10 mins stirring, the reaction mixture was heated to reflux overnight. The color of the reaction change from yellow to brown. Yellow precipitate appeared after 1 hours refluxing. After the reaction finished, the precipitate was collected, washed with methanol and

recrystallized with acetonitrile, then purified with semi-preparative HPLC to give the product as orange powder 253.7 mg (yield: 65%). ^{1}H NMR (400 MHz, dmso) δ 11.79 (s, 1H), 8.60 (d, *J* = 6.8 Hz, 2H), 8.17 (d, *J* = 6.8 Hz, 2H), 8.10 (d, *J* = 16.0 Hz, 1H), 7.87 (s, 1H), 7.24 (d, *J* = 8.1 Hz, 1H), 7.14 (d, *J* = 16.0 Hz, 1H), 6.98 (d, *J* = 8.2 Hz, 1H), 4.13 (s, 3H), 2.61 (s, 3H), 2.43 (s, 3H). ^{13}C NMR (101 MHz, dmso) δ 154.91, 144.59, 144.16, 136.07, 135.01, 130.49, 126.22, 124.04, 121.77, 120.47, 115.62, 111.67, 110.30, 46.47, 21.81, 12.48. MS(ESI$^+$): 263.1 (for calculated $C_{18}H_{19}N_2^+$ 263.2)

9. Synthesis of compound **10**, **5-I-7-FF16**

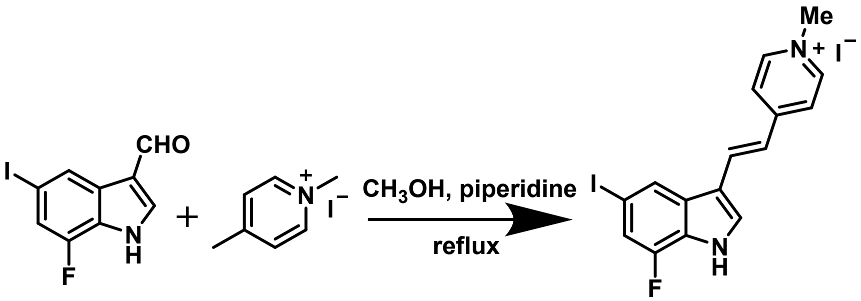

Put 258.6 mg 1,4-dimethylpyridium iodide (1.1 mmol) and 289.0 mg 2-chloro-1*H*-indole-3-carbaldehyde (1.0 mmol) into 10 mL methanol. Stirred for 5 mins, then added 19.7 μL piperidine (17 mg, 0.2 mmol). After 10 mins stirring, the reaction mixture was heated to reflux overnight. The color of the reaction change from yellow to brown. Yellow precipitate appeared after 1 hours refluxing. After the reaction finished, semi-preparative HPLC were used to purify the product. With freeze-dried, the product was gotten as orange powder 202.4 mg (yield: 40%). ^{1}H NMR (400 MHz, dmso) δ 12.57 (s, 1H), 8.71 (d, *J* = 6.8 Hz, 2H), 8.30 (d, *J* = 1.3 Hz, 1H), 8.21 (dd, *J* = 16.3, 1.9 Hz, 1H), 8.13 (d, *J* = 6.7 Hz, 2H), 8.03 (d, *J* = 3.2 Hz, 1H), 7.41 (d, *J* = 10.2 Hz, 1H), 7.28 (d, *J* = 16.3 Hz, 1H), 4.17 (d, *J* = 3.6 Hz, 3H). ^{13}C NMR (101 MHz, dmso) δ 154.09, 150.59, 148.10, 144.83, 134.72, 132.11, 131.27, 125.10, 122.54, 118.97, 116.67, 113.90, 84.25, 46.90. MS(ESI$^+$): 379.0 (for calculated $C_{16}H_{13}FIN_2^+$ 379.0)

10. Synthesis of compound **11**, **NO$_2$F16**

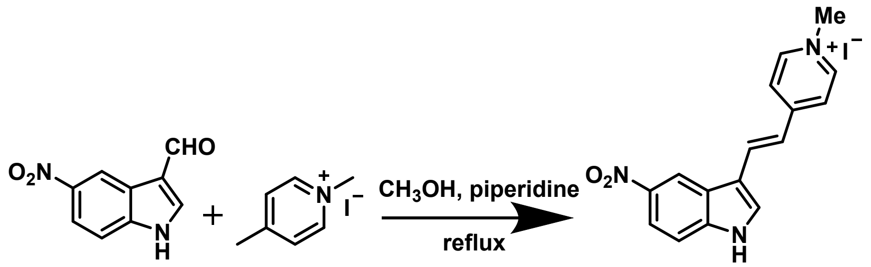

Put 258.6 mg 1,4-dimethylpyridium iodide (1.1 mmol) and 190.2 mg 2-chloro-1*H*-indole-3-carbaldehyde (1.0 mmol) into 10 mL methanol. Stirred for 5 mins, then added 19.7 μL piperidine (17 mg, 0.2 mmol). After 10 mins stirring, the reaction mixture was heated to reflux overnight. The color of the reaction change from yellow to brown. Yellow precipitate appeared after 1 hours refluxing. After the reaction finished, the precipitate was collected, washed with methanol and recrystallized with acetonitrile, then purified with semi-preparative HPLC to give the product as orange powder 285.0 mg (yield: 70%). ^{1}H NMR (400 MHz, dmso) δ 12.41 (s, 1H), 8.96 (d, *J* = 3.0 Hz, 1H), 8.79 – 8.68 (m, 2H), 8.36 – 8.19 (m, 2H), 8.14 (s, 2H), 8.03 (d, *J* = 8.8 Hz, 1H), 7.60 (d, *J* = 8.7 Hz, 1H), 7.29 (d, *J* = 16.2 Hz, 1H), 4.19 (s, 3H). ^{13}C NMR (101 MHz, dmso) δ 153.88, 144.83, 142.37, 140.46, 134.09, 133.19, 125.32, 122.70, 119.53, 118.30, 117.08, 115.63, 113.40, 46.97. MS(ESI^{+}): 280.1 (for calculated $C_{16}H_{14}N_{3}O_{2}^{+}$ 280.1)

11. Synthesis of **5-Cl-7-MeF16**

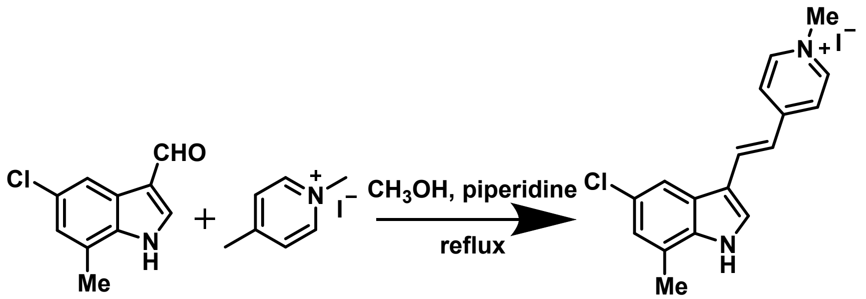

Put 258.6 mg 1,4-dimethylpyridium iodide (1.1 mmol) and 193.6 mg 5-chloro-7-methyl-1*H*-indole-3-carbaldehyde (1.0 mmol) into 10 mL methanol. Stirred for 5 mins, then added 19.7 μL piperidine (17 mg, 0.2 mmol). After 10 mins stirring, the reaction mixture was heated to reflux overnight. The color of the reaction change from yellow to brown. Yellow precipitate appeared after 1.5 hours refluxing. After the reaction finished, the precipitate was collected, washed with

methanol and recrystallized with acetonitrile, then purified with semi-preparative HPLC to give the product as orange powder 205.0 mg (yield: 50%). ^{1}H NMR (400 MHz, dmso) δ 12.13 (s, 1H), 8.73 (d, J = 6.6 Hz 2H), 8.24 (d, J = 16.3 Hz, 1H), 8.14 (d, J = 6.7 Hz, 2H), 8.06 (d, J = 10.1 Hz 2H), 7.30 (d, J = 16.3 Hz, 1H), 7.10 (s, 1H), 4.20 (s, 3H), 2.50 (s, 3H). ^{13}C NMR (126 MHz, dmso) δ 154.47, 144.74, 135.78, 135.73, 132.51, 126.40, 126.27, 124.37, 123.61, 122.30, 117.94, 117.38, 114.05, 46.83, 16.93. MS(ESI^{+}): 283.1 (for calculated $C_{17}H_{16}ClN_2^{+}$ 327.1)

NMR spectrum

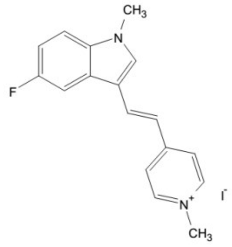

^{1}H NMR (400 MHz, dmso) δ 8.68 (d, J = 6.7 Hz, 2H), 8.19 – 8.10 (m, 4H), 7.94 (s, 1H), 7.46 (dd, J = 10.0, 2.2 Hz, 1H), 7.25 (d, J = 16.3 Hz, 1H), 7.11 (td, J = 9.3, 2.3 Hz, 1H), 4.16 (s, 3H), 3.83 (s, 3H).

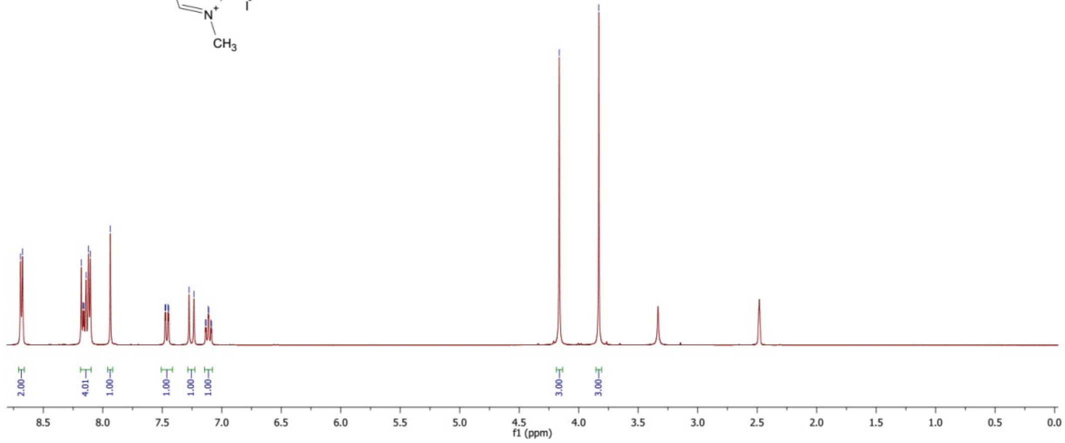

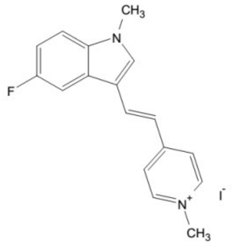

^{13}C NMR (101 MHz, dmso) δ 161.14, 158.78, 154.35, 144.69, 138.81, 138.68, 136.39, 135.45, 122.24, 117.74, 113.14, 110.04, 109.80, 98.30, 98.03, 46.78, 33.73.

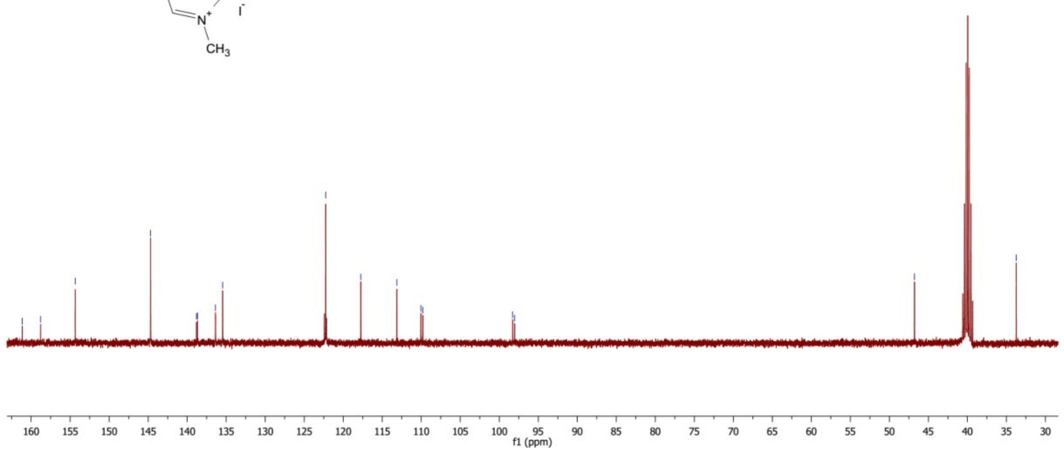

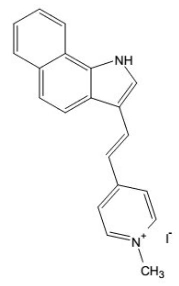

^{1}H NMR (400 MHz, dmso) δ 12.77 (s, 1H), 8.69 (d, *J* = 6.9 Hz, 2H), 8.39 (d, *J* = 8.2 Hz, 1H), 8.31 (d, *J* = 16.3 Hz, 1H), 8.25 (d, *J* = 8.7 Hz, 1H), 8.14 (d, *J* = 6.9 Hz, 2H), 8.07 (d, *J* = 2.8 Hz, 1H), 7.99 (d, *J* = 8.0 Hz, 1H), 7.68 (d, *J* = 8.8 Hz, 1H), 7.59 (dd, *J* = 11.2, 4.0 Hz, 1H), 7.47 (dd, *J* = 11.1, 4.0 Hz, 1H), 7.38 (d, *J* = 16.3 Hz, 1H), 4.17 (s, 3H).

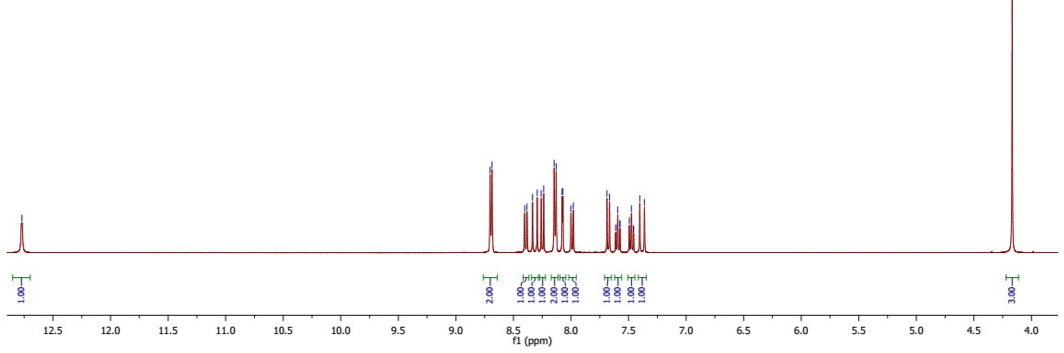

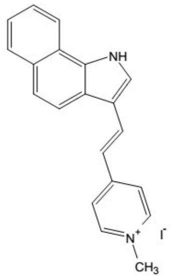

^{13}C NMR (101 MHz, dmso) δ 154.39, 144.72, 136.21, 132.52, 130.60, 129.13, 128.85, 126.46, 124.96, 122.33, 122.29, 122.02, 121.74, 121.19, 120.24, 118.15, 115.56, 46.80.

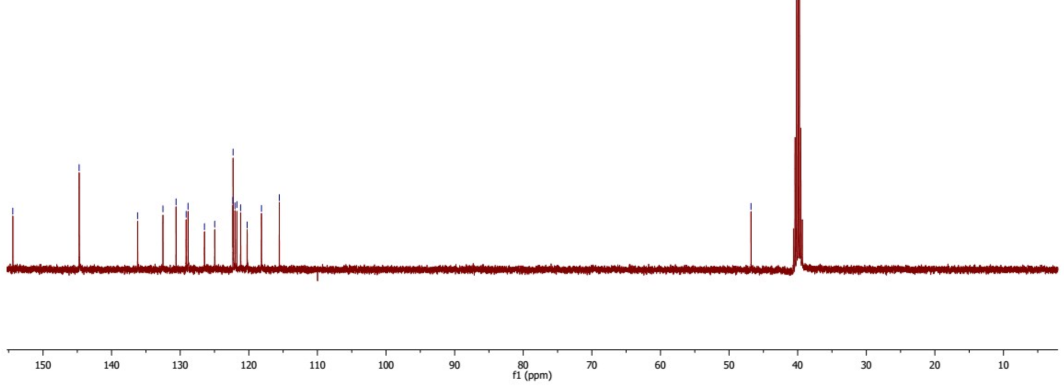

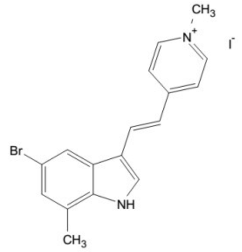

^{1}H NMR (400 MHz, dmso) δ 12.07 (s, 1H), 8.69 (s, 2H), 8.21 (d, *J* = 16.3 Hz, 1H), 8.12 (d, *J* = 4.5 Hz, 3H), 8.03 (s, 1H), 7.26 (d, *J* = 16.3 Hz, 1H), 7.18 (s, 1H), 4.17 (s, 3H), 2.47 (s, 3H).

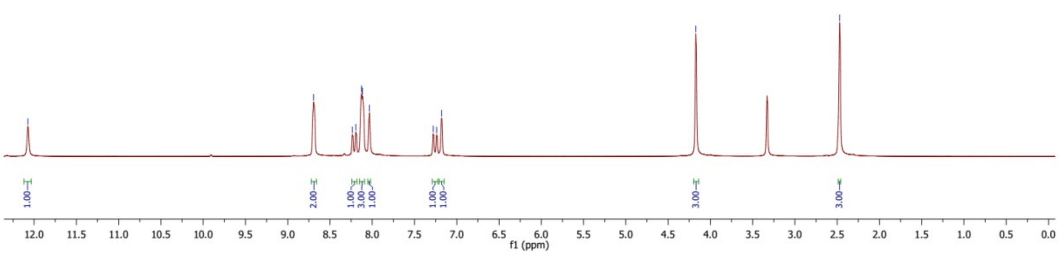

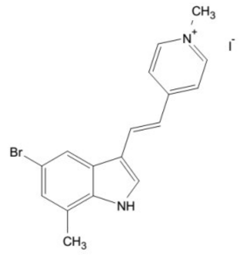

^{13}C NMR (101 MHz, dmso) δ 154.38, 144.67, 135.97, 135.61, 132.14, 127.02, 126.10, 124.73, 122.27, 120.25, 117.95, 114.37, 113.89, 46.80, 16.85.

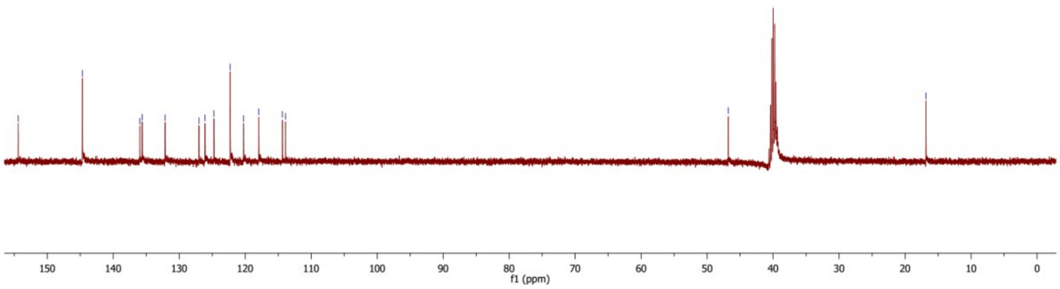

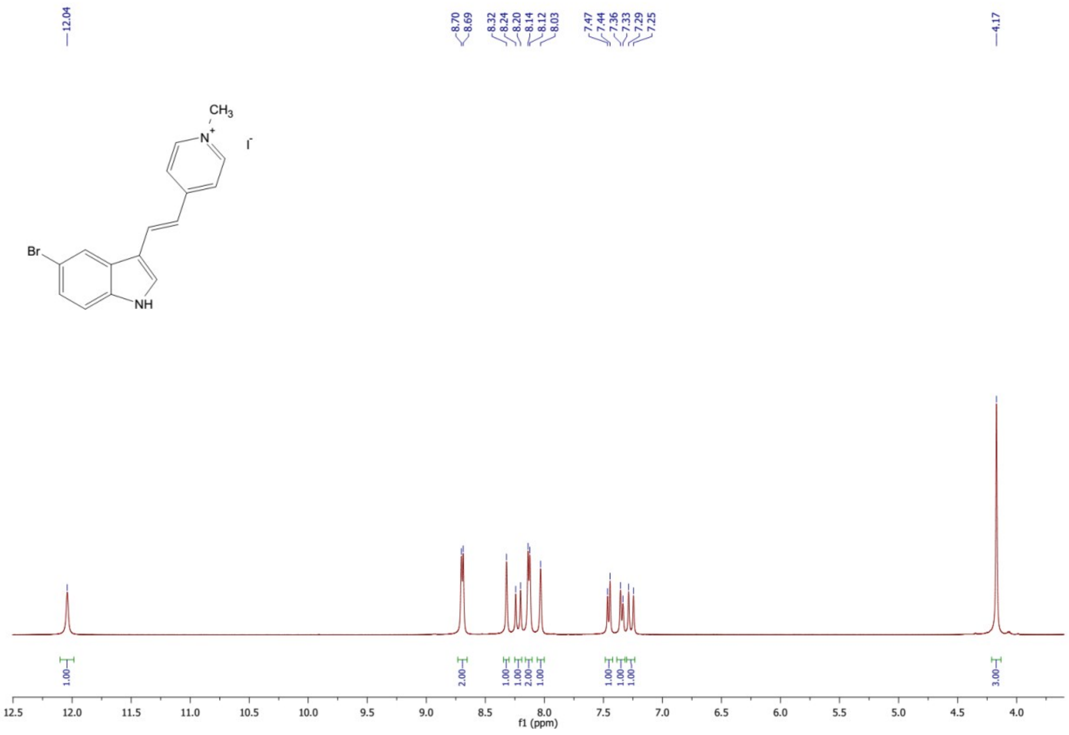

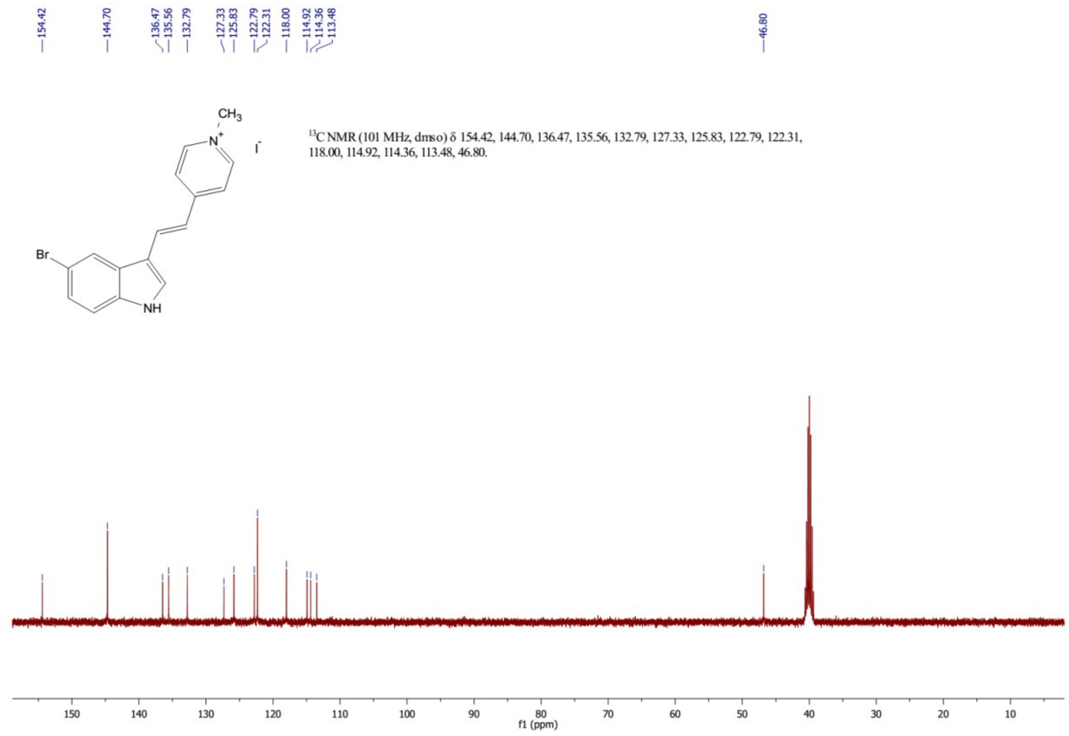

^{13}C NMR (101 MHz, dmso) δ 154.42, 144.70, 136.47, 135.56, 132.79, 127.33, 125.83, 122.79, 122.31, 118.00, 114.92, 114.36, 113.48, 46.80.

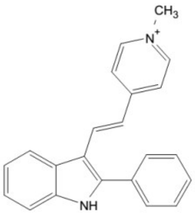

^{1}H NMR (400 MHz, dmso) δ 12.23 (s, 1H), 8.62 (d, J = 5.8 Hz, 2H), 8.22 (d, J = 7.7 Hz, 1H), 8.04 (d, J = 5.7 Hz, 2H), 7.93 (d, J = 16.0 Hz, 1H), 7.68 (d, J = 6.8 Hz, 2H), 7.62 (t, J = 7.0 Hz, 2H), 7.56 (d, J = 7.1 Hz, 1H), 7.51 (d, J = 7.7 Hz, 1H), 7.42 (d, J = 16.1 Hz, 1H), 7.29 (dd, J = 16.9, 8.0 Hz, 2H), 4.16 (s, 3H).

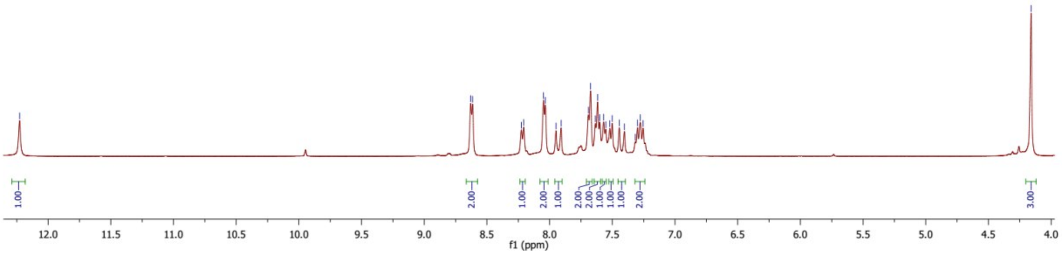

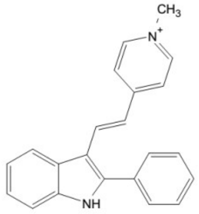

^{13}C NMR (101 MHz, dmso) δ 154.32, 144.62, 144.30, 137.39, 135.60, 131.32, 130.06, 129.63, 129.57, 126.06, 123.82, 122.36, 121.97, 121.39, 118.96, 112.75, 110.41, 46.80.

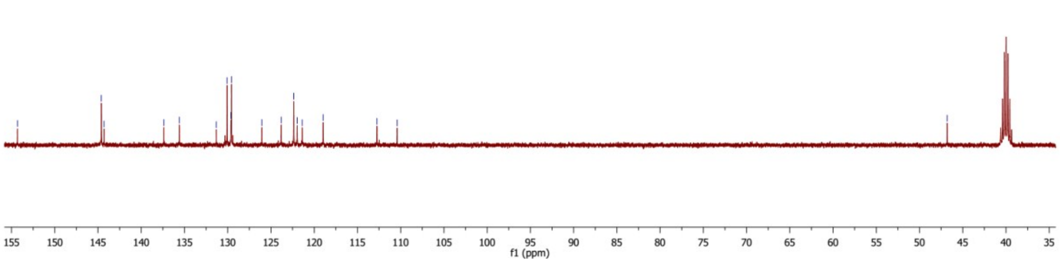

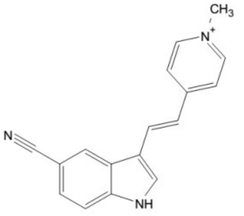

^{1}H NMR (400 MHz, dmso) δ 12.29 (s, 1H), 8.74 (d, *J* = 5.5 Hz, 2H), 8.69 (s, 1H), 8.23 (d, *J* = 16.4 Hz, 1H), 8.15 (s, 3H), 7.61 (dd, *J* = 29.8, 8.2 Hz, 2H), 7.38 (d, *J* = 16.3 Hz, 1H), 4.19 (s, 3H).

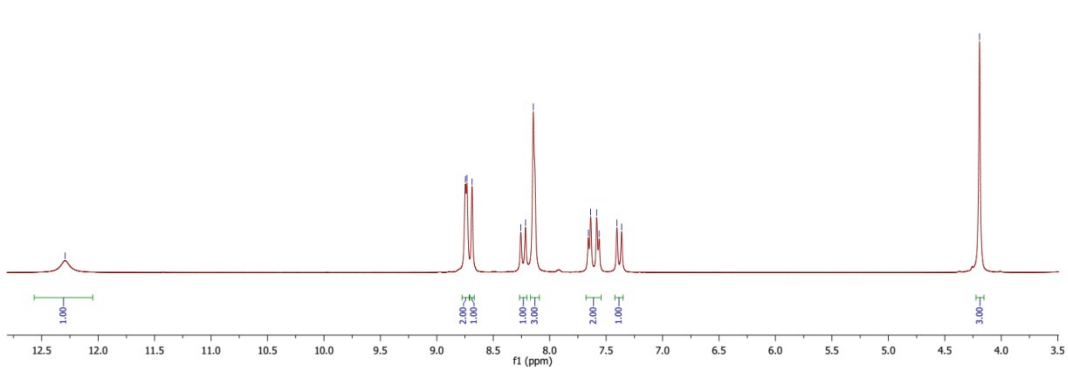

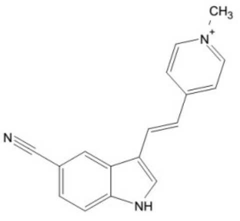

^{13}C NMR (101 MHz, dmso) δ 154.21, 144.86, 139.51, 134.81, 133.71, 126.10, 125.99, 125.25, 122.52, 120.83, 119.14, 114.35, 114.24, 103.52, 46.95.

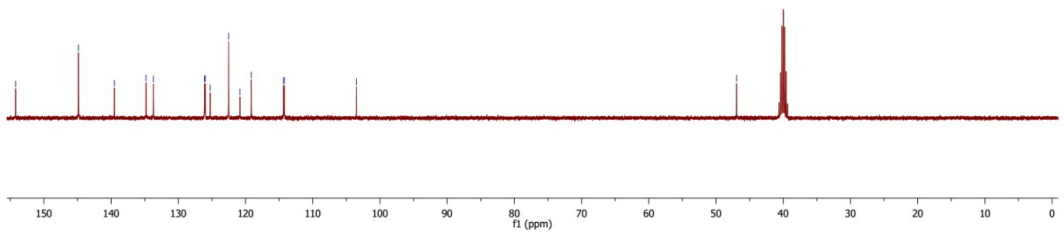

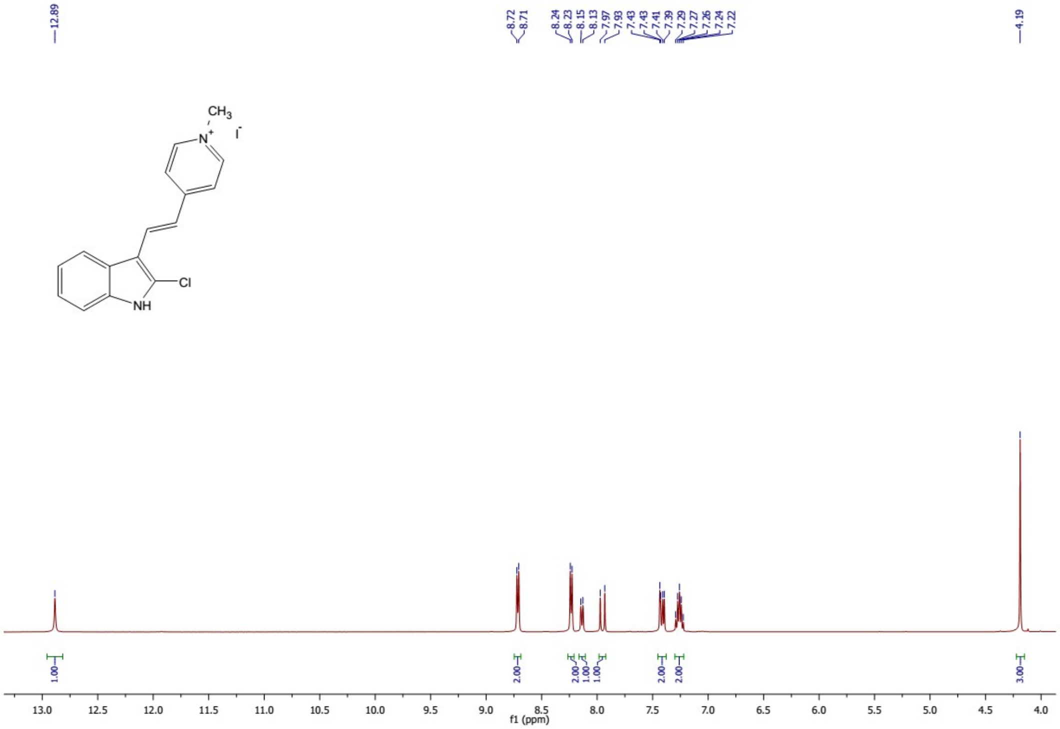
12.89
8.72
8.71
8.24
8.23
8.15
8.13
7.97
7.93
7.43
7.41
7.39
7.29
7.27
7.26
7.24
7.22
4.19
CH3
N+
I-
Cl
NH
1.00
2.00
2.00
1.00
1.00
2.00
2.00
3.00
f1 (ppm)

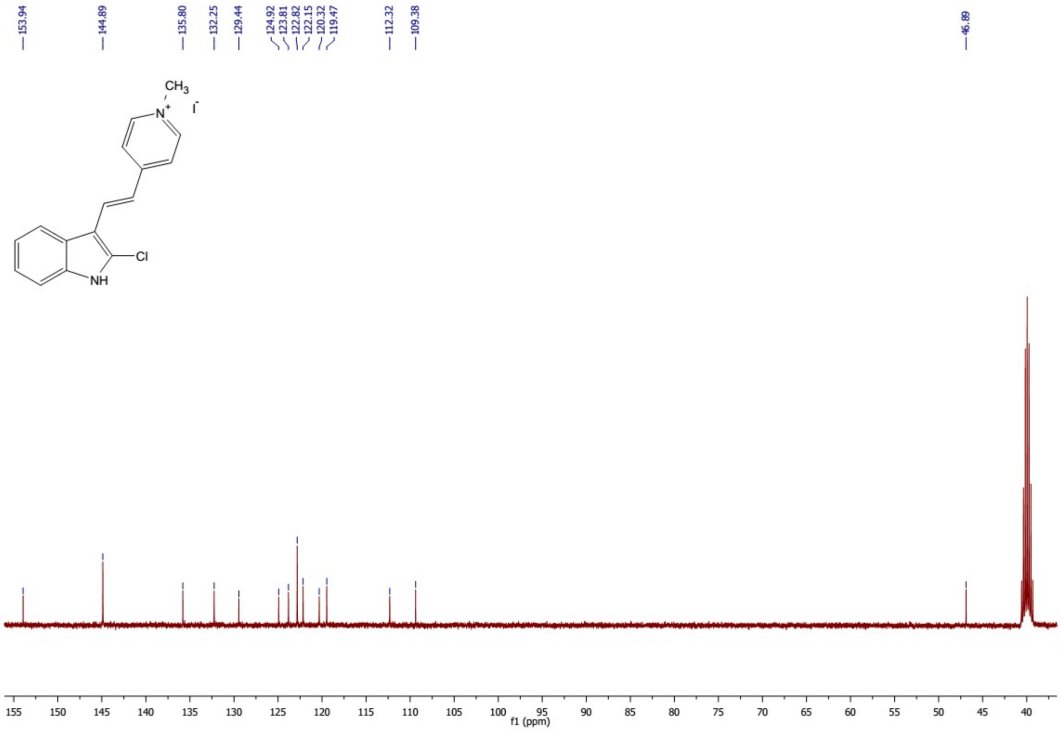
153.94
144.89
135.80
132.25
129.44
124.92
123.81
122.82
122.15
120.32
119.47
112.32
109.38
46.89
CH3
N+
I-
Cl
NH
f1 (ppm)

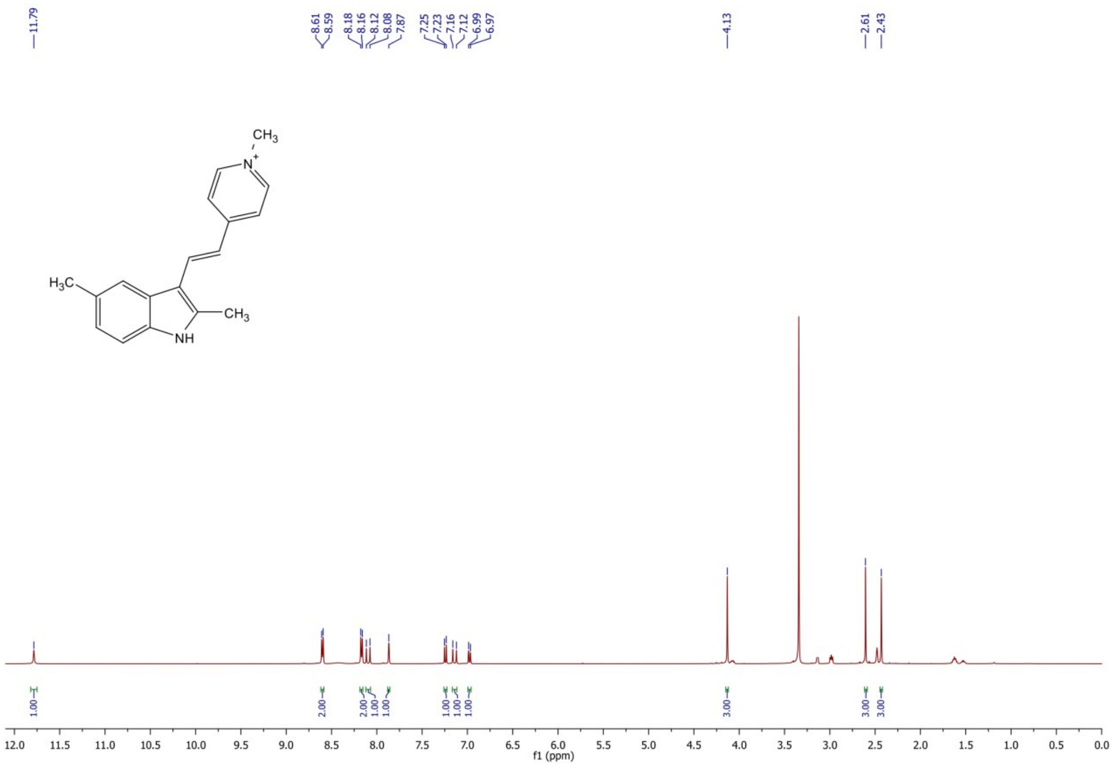
11.79
8.61
8.59
8.18
8.16
8.12
8.08
7.87
7.25
7.23
7.16
7.12
6.99
6.97
4.13
2.61
2.43
CH3
N+
H3C
CH3
NH
1.00
2.00
2.00
1.00
1.00
1.00
1.00
1.00
3.00
3.00
3.00
f1 (ppm)

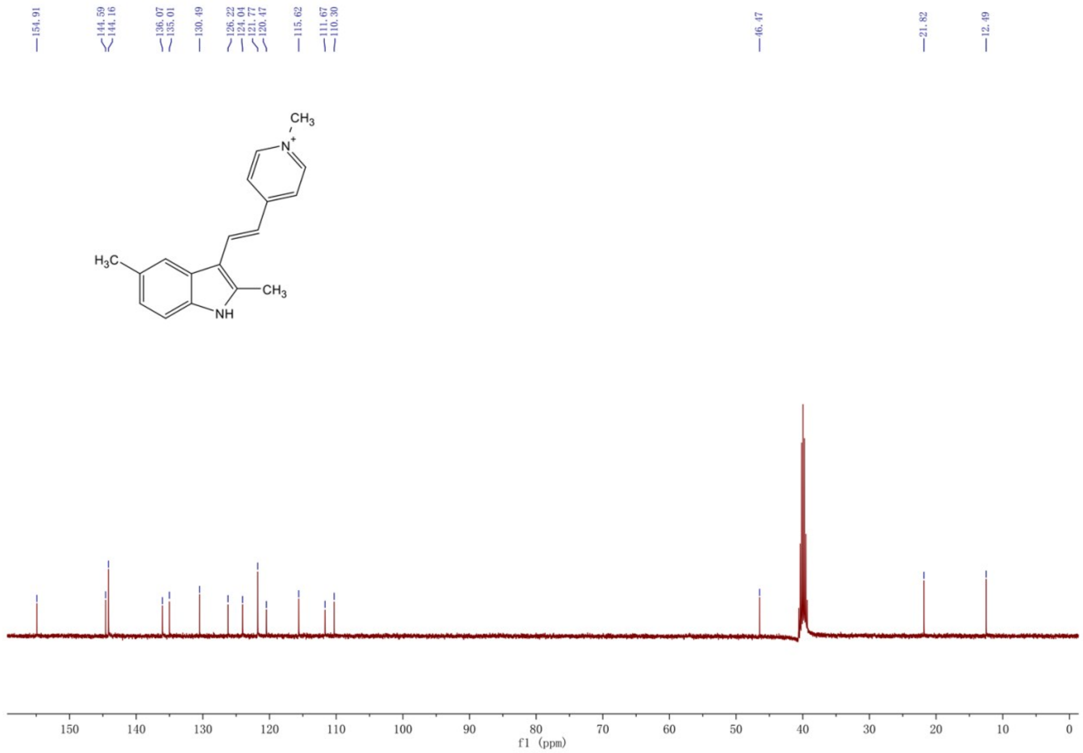
154.91
144.59
144.16
136.07
135.01
130.49
126.22
124.04
121.77
120.47
115.62
111.67
110.30
46.47
21.82
12.49
CH3
N+
H3C
CH3
NH
f1 (ppm)

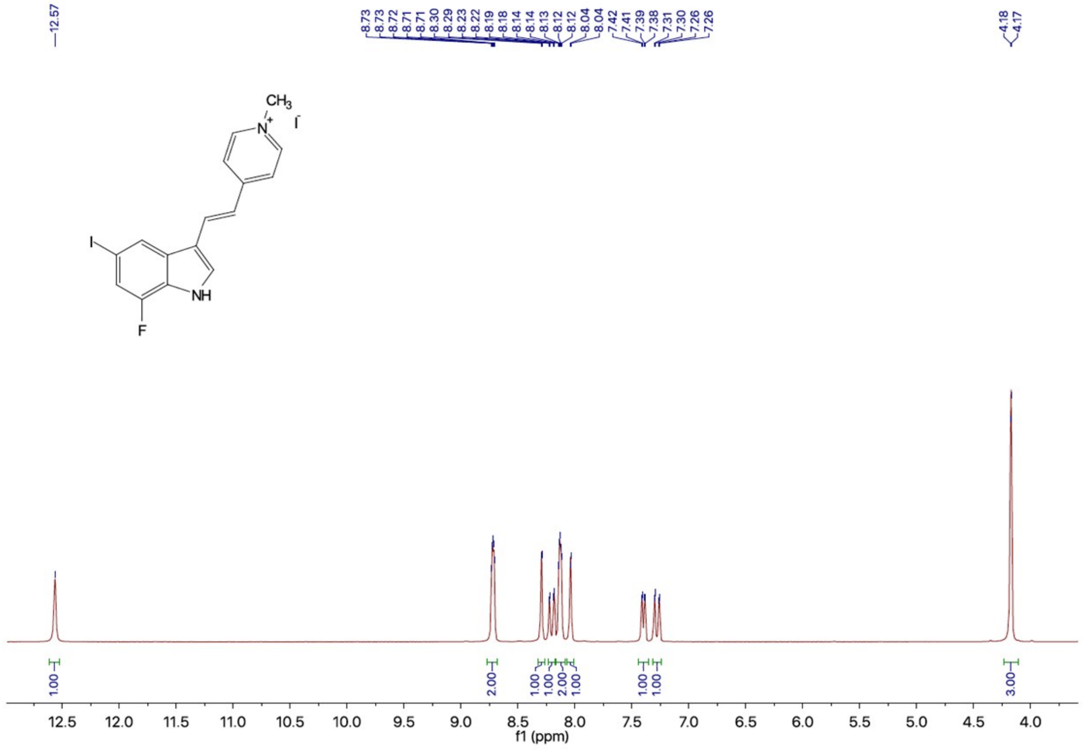

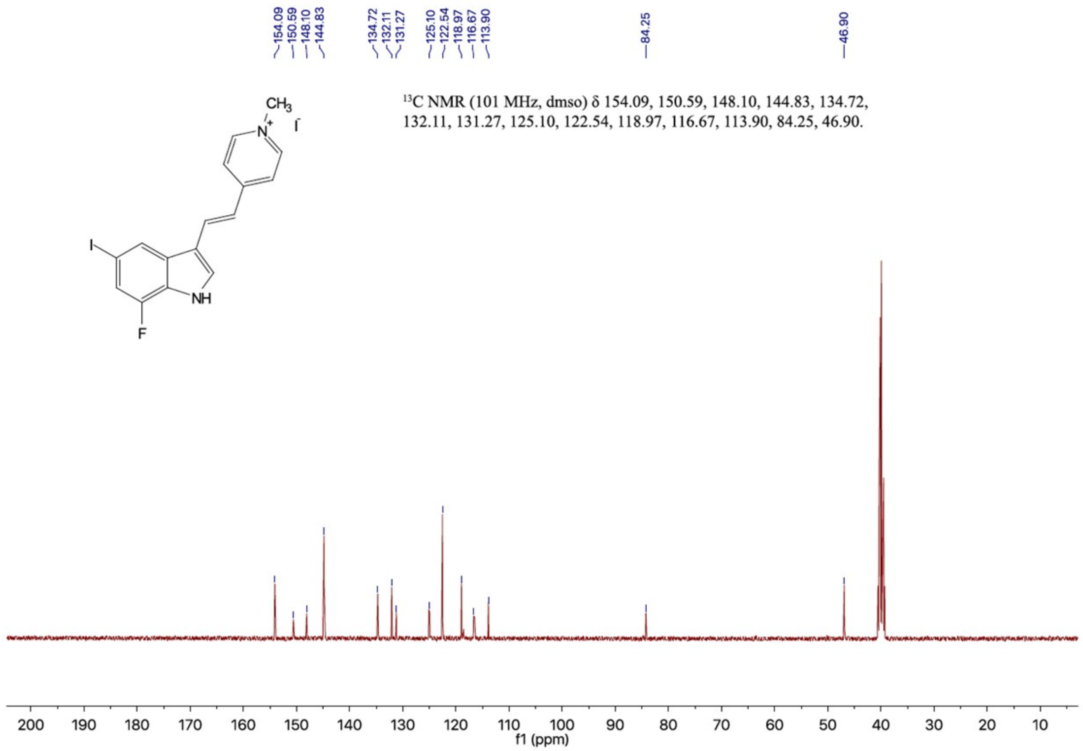

^{13}C NMR (101 MHz, dmso) δ 154.09, 150.59, 148.10, 144.83, 134.72, 132.11, 131.27, 125.10, 122.54, 118.97, 116.67, 113.90, 84.25, 46.90.

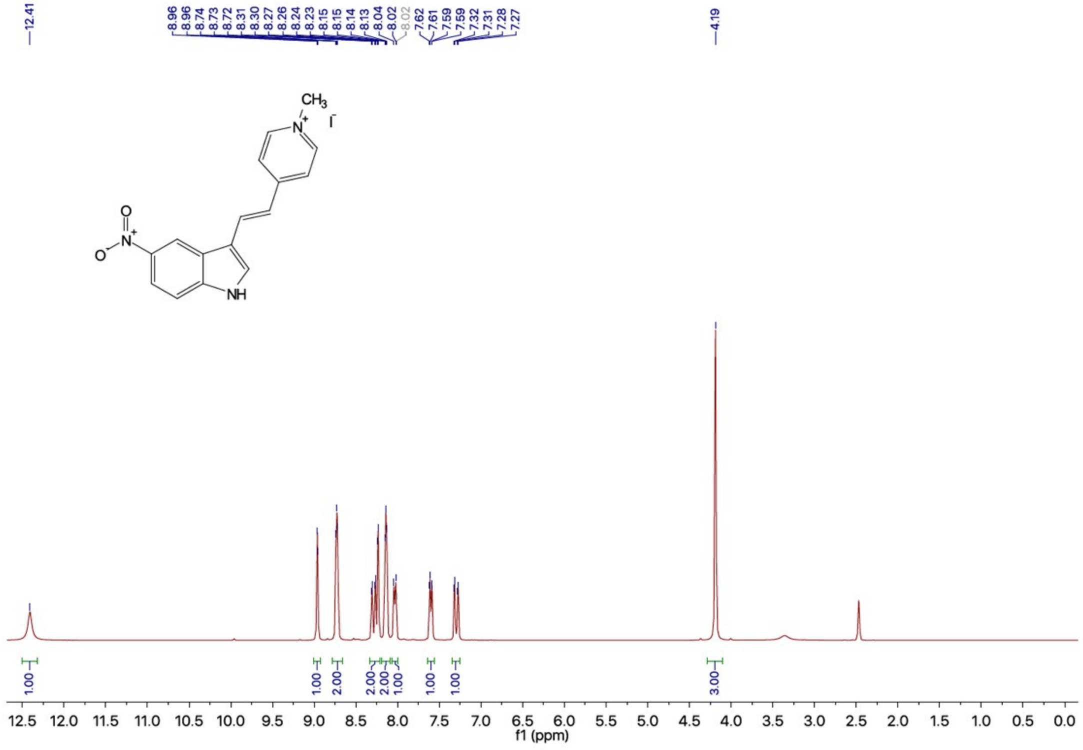

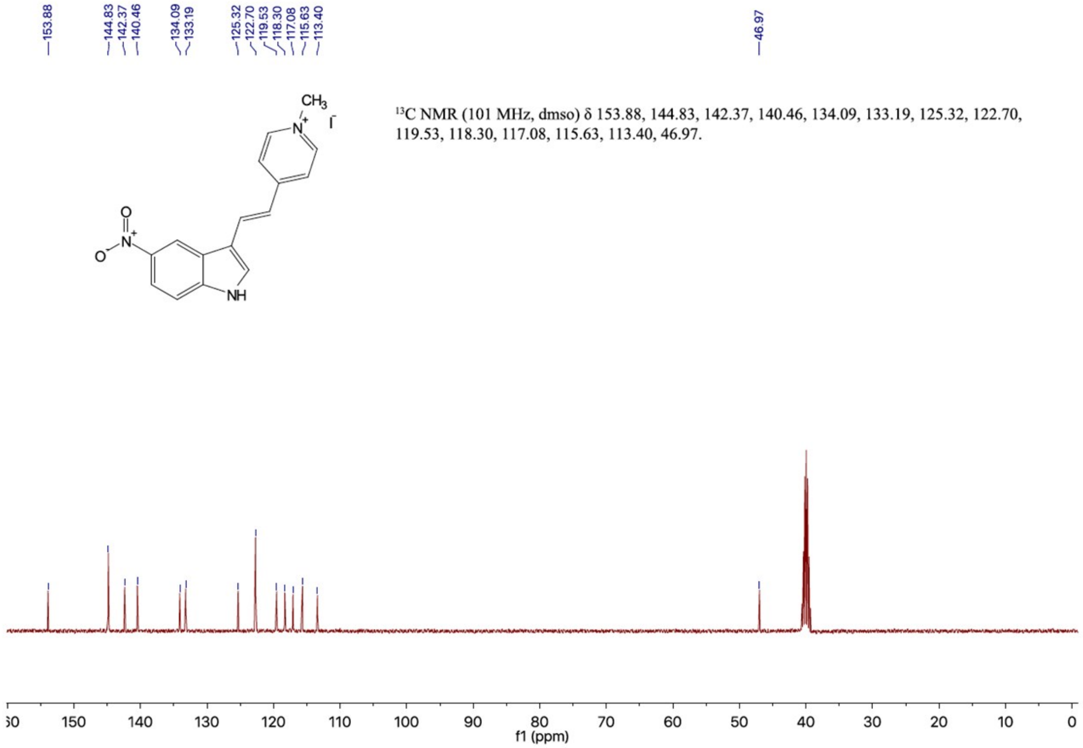

^{13}C NMR (101 MHz, dmso) δ 153.88, 144.83, 142.37, 140.46, 134.09, 133.19, 125.32, 122.70, 119.53, 118.30, 117.08, 115.63, 113.40, 46.97.

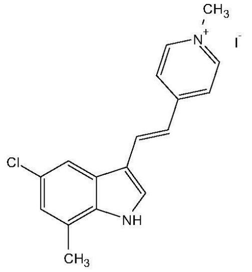

^{1}H NMR (400 MHz, DMSO) δ 12.13 (s, 1H), 8.73 (d, J = 6.6 Hz, 2H), 8.24 (d, J = 16.3 Hz, 1H), 8.14 (d, J = 6.7 Hz, 2H), 8.06 (d, J = 10.1 Hz, 2H), 7.30 (d, J = 16.3 Hz, 1H), 7.10 (s, 1H), 4.20 (s, 3H), 2.50 (s, 3H).

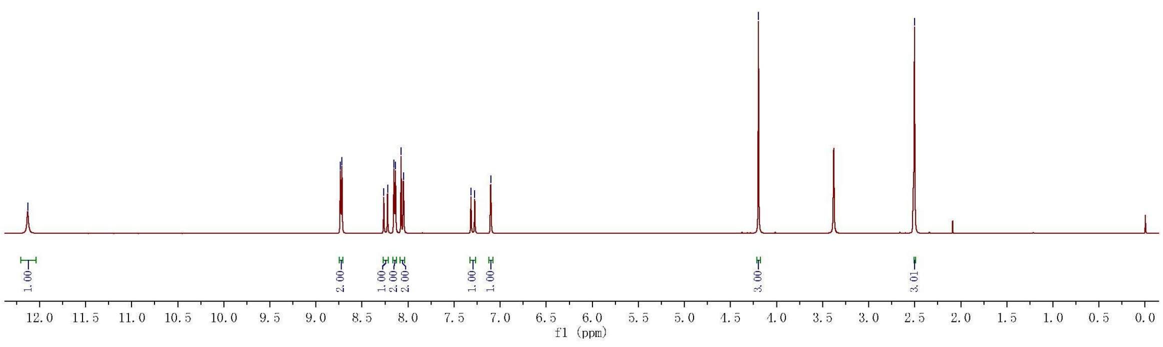

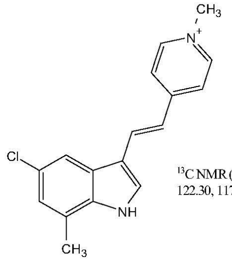

^{13}C NMR (126 MHz, DMSO) δ 154.47, 144.74, 135.78, 135.73, 132.51, 126.40, 126.27, 124.37, 123.61, 122.30, 117.94, 117.38, 114.05, 46.83, 16.93.

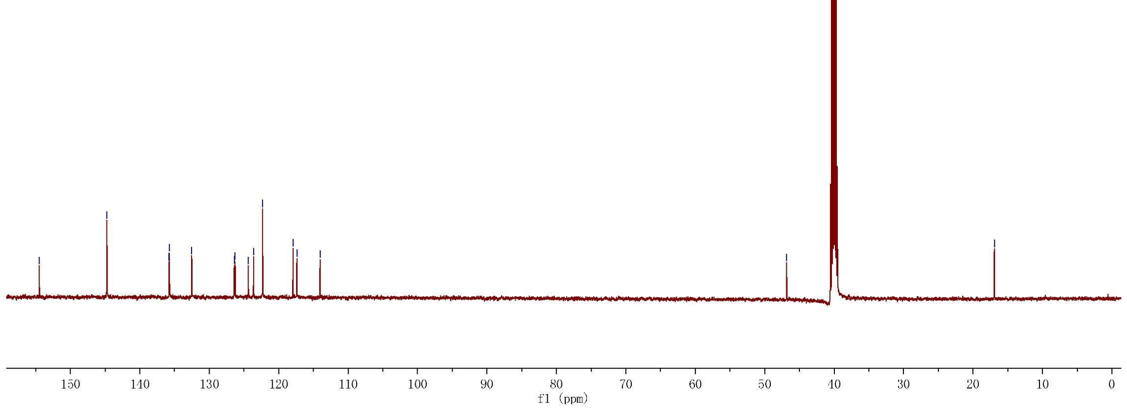

References

[1] S. Wu, Q. Cao, X. Wang, K. Cheng, Z. Cheng, *Chem. Commun.* **2014**, *50*, 8919-8922.

[2] R. A. Velapoldi, H. H. Tonnesen, *Journal of Fluorescence* **2004**, *14*, 465-472.
